# Game-based Sprint retrospectives: multiple action research

**DOI:** 10.1007/s10664-021-10043-z

**Published:** 2021-10-19

**Authors:** Adam Przybyłek, Marta Albecka, Olga Springer, Wojciech Kowalski

**Affiliations:** 1 Faculty of Electronics, Telecommunications and Informatics, Gdansk University of Technology, Narutowicza 11/12, 80-233 Gdańsk, Poland; 2 Dynatrace Sp. z o.o, Aleja Grunwaldzka 411, 80-309 Gdańsk, Poland; 3 OKE Poland Sp. z o.o, Jana Heweliusza 11, 80-890 Gdańsk, Poland

**Keywords:** Collaborative games, Serious games, Retrospective, Agile, Scrum, Software process improvement, Team communication

## Abstract

In today’s fast-paced world of rapid technological change, software development teams need to constantly revise their work practices. Not surprisingly, regular reflection on how to become more effective is perceived as one of the most important principles of Agile Software Development. Nevertheless, running an effective and enjoyable retrospective meeting is still a challenge in real environments. As reported by several studies, the Sprint Retrospective is an agile practice most likely to be implemented improperly or sacrificed when teams perform under pressure to deliver. To facilitate the implementation of the practice, some agile coaches have proposed to set up retrospective meetings in the form of retrospective games. However, there has been little research-based evidence to support the positive effects of retrospective games. Our aim is to investigate whether the adoption of retrospective games can improve retrospective meetings in general and lead to positive societal outcomes. In this paper, we report on an Action Research project in which we implemented six retrospective games in six Scrum teams that had experienced common retrospective problems. The received feedback indicates that the approach helped the teams to mitigate many of the “accidental difficulties” pertaining to the Sprint Retrospective, such as lack of structure, dullness, too many complaints, or unequal participation and made the meetings more productive to some degree. Moreover, depending on their individual preferences, different participants perceived different games as having a positive impact on their communication, motivation-and-involvement, and/or creativity, even though there were others, less numerous, who had an opposite view. The advantages and disadvantages of each game as well as eight lessons learned are presented in the paper.

## Introduction

Over the last two decades, we have seen a major change in how software is developed with the adoption of agile methods. Agile methods embrace the fact that software development projects are too complex to be included in a full-scale plan, and the requirements cannot be fully understood or specified up front because they are rarely ready to be collected (Boehm et al. 2001; Campbell et al. 2016). Thereby, agile methods originated from the practice of encouraging close collaboration among the development team and between the team and the customer, to accommodate changes, and to work in rapid, iterative development cycles.

Due to their positive impact on productivity, agile methods have become mainstream in the software industry (Cao et al. 2009; Mundra et al. 2013; Eloranta et al. 2016; Schön et al. 2017; Gaikwad et al. 2019; Butt et al. 2019; Jarzębowicz and Sitko 2019; Küpper et al. 2019). Nevertheless, despite numerous agile transformation success stories, most organizations struggle to fully implement an agile method (VersionOne, 2020). Indeed, agile methods are claimed to be easy to understand (Schwaber 2004; López-Martínez et al. 2016), but hard to follow in practice, which has been confirmed even by their inventors. For instance, according to Beck (2004) “XP is simple in its details, but it is hard to execute,” while Schwaber and Sutherland (2017) describes Scrum as “simple to understand, (but) difficult to master.”

When companies encounter challenges in adopting agile practices, the general tendency is to abandon the practice (Yu and Petter 2014). Yet, when practices are abandoned, neglected, or compromised, a company fails to take full advantage of the method. Fortunately, agile teams are equipped with a mechanism for self-repair, which is called Retrospective (Highsmith and Fowler 2001). Unfortunately, this practice itself is often implemented improperly due to little guidance from agile methods. For instance, XP does not mention Retrospective at all, while neither Scrum nor Kanban describe specific procedure for running this meeting. Accordingly, in many projects, retrospectives are conducted in a mechanical way, without an understanding of the real value of the ceremony (Mas et al. 2018), while agile teams have difficulties in transforming the lessons learned into action (Drury et al. 2012; Dybå et al. 2014b; Andriyani et al. 2017). What is worse, if retrospective meetings are repeated in the same way over and over again, they may get dull, which demotivates team members and negatively affects outcomes. Consequently, team members start to see retrospectives as a waste of time and they stop attending them (Gonçalves and Linders 2014). As agile teams usually work under intense time pressure (project managers often believe that Scrum teams can do “twice the work in half the time” (Sutherland 2015)), the temptation to skip a nonproductive retrospective is even stronger (Moe et al. 2012; Hoda et al. 2013; Babb et al. 2014; Dybå et al. 2014a; Khanna, 2018). Indeed, many researchers and practitioners have reported that retrospective meetings are skipped because “the same old things come up every time instead of insightful ideas” or the team “has run out of things to improve” (Lamoreux 2005; Nikitina et al. 2012; Jeffries 2013; Carlson 2013; Eloranta et al. 2016; Drægert and Petersen 2016; Przybyłek and Kotecka 2017). Furthermore, several other problems that frequently prevent teams from conducting productive retrospectives have been brought up (Lamoreux 2005; Gonçalves and Linders 2014; Derby and Larsen 2006; Kua 2013; Loeffler 2017; Ringstad et al. 2011; Rubin 2012):

- the lack of meeting structure narrows down the discussion;
- a few vocal participants dominate the discussion, while others only listen even though they have profound views on things;
- participants use the meeting primarily to complain rather than to improve.

In the meantime, the usage of game elements in non-gaming practices has become an emerging subject for improving the software development processes (Yilmaz et al. 2016; Yilmaz and O’Connor 2016; Stettina et al. 2018). It has been evidenced that turning an activity into a game may address a range of problems in software engineering, including poor communication within the development team, inadequate teamwork, lack of motivation, and boredom with work (Olgun et al. 2017; Akarsu et al. 2018; Üsfekes et al. 2019; Yilmaz et al. 2019; Daylamani-Zad et al. 2020). Ipso facto, to mitigate common retrospective problems, some agile coaches have proposed setting up retrospective meetings in the form of games, which are claimed to break the habitual routine and enforce the structure of the meeting (Roden and Williams 2015). However, there has been little research to date on how retrospective games affect retrospective meetings (Matthies and Dobrigkeit 2021). In our pilot study (Przybyłek and Kotecka 2017), we successfully implemented game-based retrospectives in 3 teams in Intel Technology Poland, but we failed to meet scientific rigor. The teams stated that retrospective games made their retrospectives more engaging, insightful and broke the monotony. Encouraged by the preliminary results, in this paper, we explore retrospective games further and repeat the study in three new companies to make it more robust. Our aim is to investigate whether the adoption of retrospective games can improve retrospective meetings in general and lead to positive societal outcomes.

Since we need an industrial context to embed our research, we are carrying out the project as Action Research (Baskerville and Myers 2004). Action Research is a method for co-development of research results, where academia and industry work together to solve problematic situations. Through this co-development, the academics and practitioners learn from each other, and thus they develop research results which contribute to both the industrial practice and scientific knowledge development (Staron 2020).

As a result of this work, four major contributions can be enumerated, as follows:

- a proposal of a generic model of running a retrospective game, which is based on the group creativity model (Nijstad and Paulus 2003) as well as the state-of-the-art in group idea generation;
- a proposal of a systematic methodological approach to choose and adopt retrospective games that suit the Scrum team the best and have a chance to improve their retrospective practice;
- an in-depth discussion of the promises and realities of game-based retrospectives, including the advantages and disadvantages of six implemented games as well as eight lessons learned that were identified during our Action Research project;
- a refinement of the Action Research methodology, which consists of a revised validity system for Action Research studies in software engineering, and a proposal of a new research design borrowed from Case Study.

The remainder of this paper is structured as follows. The next section outlines the theoretical and practical background on an agile retrospective, team creativity, and collaborative games. In Section 3, we present related work. In Section 4, the research method, questions, context, as well as data collection and analysis details are discussed. This is followed by the core section of this paper in which we report on the conducted Action Research cycle, interpret the collected data, and provide the lessons learned. In Section 6, we elaborate on the threats to validity, while in Section 7, we consider the implications of our research. Section 8 discusses the rigor and the relevance of the project. Finally, we summarize the key findings and suggest directions for future research.

## Theoretical and Practical Background

This section introduces the main concepts that must be understood in the context of our work: agile retrospective, team creativity, serious games, and retrospective games.

### Agile retrospective

One of the core principles of all agile methods is the need for continuous process improvement: “At regular intervals, the team reflects on how to become more effective, then tunes and adjusts its behavior accordingly” (Highsmith and Fowler 2001). An agile practice that provides time and space for the team to come together in order to inspect, fix, and improve their process is a retrospective meeting. The Scrum framework calls this the Sprint Retrospective (Schwaber and Sutherland 2020). However, team reflexivity as a group level construct is older than the Agile Manifesto (for review, see (Collier et al. 1996; Dingsøyr and Hanssen 2003)). It has been defined as a social-cognitive process, in which “team members collectively reflect upon the team’s objectives, strategies, and processes as well as their wider organization and environments, and adapt them accordingly” (West, 1996). In fact, systematic reflection as a prominent tool for learning from experience is an important determinant of team effectiveness (West, 1996; Dybå et al. 2014a; Ellis et al. 2014). According to West et al. (1997), reflexive teams conduct more detailed planning, pay more attention to long-term consequences, and respond to a wider range of environmental cues.

The Sprint Retrospective is held at the end of each Sprint after the Sprint Review. All members of the team are required to attend the retrospective and actively participate in the discussion. During the meeting, three key questions should be answered (Schwaber 2004; Ringstad et al. 2011):

- What went well, that if we don’t discuss, we might forget?
- What did not work and how can we improve it?
- What will we commit to improve in the next iteration?

Accordingly, the retrospective is an opportunity for the team to look back over an iteration and recognize successes and failures; to link the related experience to people, the development process, engineering practices, and tools; and to create a plan for improvements to be enacted during the next iteration (Dybå et al. 2014b; Lehtinen et al. 2015, Lehtinen et al. 2017; Dingsøyr et al. 2018; Ilyés 2019). Indeed, the retrospective is one of the most frequently mentioned agile practices in the context of software process improvement (Mas et al. 2018; Küpper et al. 2019). The output of a retrospective are “action items” (Derby and Larsen 2006), which define identified issues as well as possible corrective actions to resolve them (Matthies 2020). All action items should be assigned to volunteers who will take appropriate steps to implement them. Anything that exceeds the authority or scope of responsibility of the team should be escalated to management and considered in an organization-level retrospective (Carlson 2013; Lehtinen et al. 2017; Guckenbiehl and Theobald 2020). The role of the management is to create an organizational context that is conducive to reflection and organizational learning (Dybå et al. 2014a). The ultimate goal of agile retrospectives is to improve productivity and work satisfaction.

### Team creativity

Creativity is the ability to come up with ideas or artifacts that are novel (i.e., original, surprising) and valuable (Amabile 1983; Sternberg 1999; Boden, 2004). It has been studied from a variety of perspectives for decades. Initially, research on creativity focused on individuals and their personality traits as the basis for creativity (Amabile 1983).

More recent studies have suggested that breakthrough ideas are rarely the result of individual effort, but emerge from the combined efforts of a number of people (Sawyer 2007). Consequently, the research has expanded to comprise team-level creativity (Hoegl & Parboteeah, 2008).

Numerous techniques have been developed to facilitate the group idea generation process (for review, see (Paulus and Nijstad 2003)). The most classic one is brainstorming (Osborn, 1953), in which group members tap their long-term memory in real-time for relevant ideas to connect to the problem being considered (Paulus and Dzindolet 2008). The rules behind brainstorming are as follows: focus on quantity (the more ideas, the better), welcome wild ideas, combine ideas and improve them, and do not be critical.

Nevertheless, contrary to Osborn’s (1957) claim that brainstorming increases the quality and quantity of ideas produced by group members, research findings have suggested the opposite effect (Taylor et al. 1958; Diehl and Stroebe 1987). Several cognitive and social processes have been considered to explain this effect, including:

- social loafing (free-riding) – the tendency of individuals to rely on the efforts of others to accomplish the task (Karau and Williams 1993);
- downward matching – the tendency of individuals to match their performance to the least productive group member (Paulus and Dzindolet 1993);
- evaluation apprehension – people often refrain from expressing ideas that they think are too controversial, weird, or unrealistic, because they fear they will receive negative evaluations from others (Diehl and Stroebe 1987);
- production blocking – only one person can speak at a time, while the other group members must sit passively waiting for their turn; in the meantime, they may forget ideas or lose their motivation to share (Nijstad et al. 2003).

To reduce the negative impact of these processes, “individual brainstorming” (also known as brainwriting) has been proposed. In brainwriting, participants silently write down their ideas on post-it notes and place them in the center of the table (Michinov 2012). Several studies have provided evidence that brainwriting groups produce more ideas than face-to-face brainstorming groups (Madsen and Finger 1978; Paulus and Brown 2003). Furthermore, a number of scholars have found that combining individual and group brainstorming brings even better results (for review, see (Korde and Paulus 2017)).

**Fig. 1:**
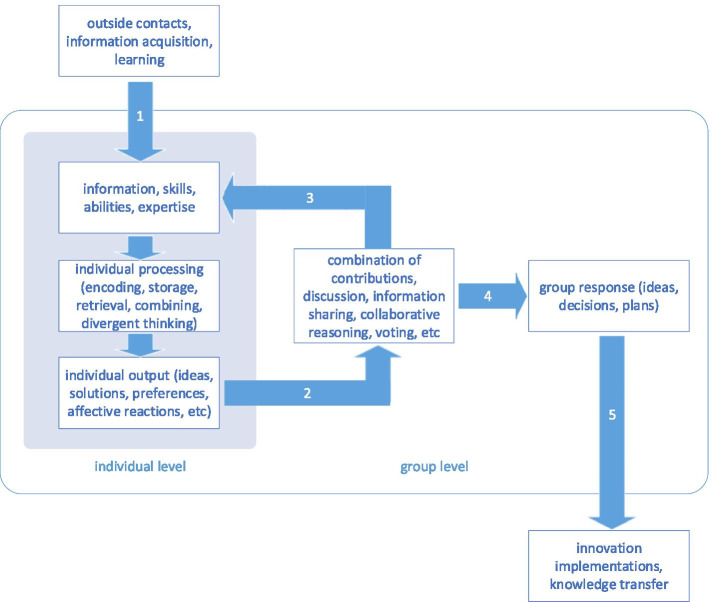
A generic model of group creativity (Nijstad and Paulus 2003)

One of the predominant group creativity models, which has influenced the literature on collective creativity for next decades (Nijstad and Stroebe 2006; Paulus and Brown 2007; De Dreu et al. 2008; Mannix 2009; Paulus and Nijstad 2019), is depicted in Fig. 1. It was proposed in 2003 by Nijstad and Paulus (2003) and it integrated different state-of-the-art cognitive models at that time (Paulus and Nijstad 2003). The shaded rectangle represents the individual’s creative process. Each group member has resources (i.e., knowledge, skills, abilities, and expertise) and is able to acquire new resources (arrow 1). These resources are used to undertake the individual processing and develop an output (ideas, solutions, etc.) on an individual level. The individual contribution is then sent (arrow 2) to the group processing (the centrally located interactions). Provided that individuals pay attention to the contributions of others, the stimulus is added to their resources (arrow 3). Individuals also receive group feedback through discussion, reasoning, and voting. The new information can be processed again on the individual level, which may result in new ideas or a shift in preferences. There is a back and forth exchange between the individual and group level until the contributions of individuals are combined to yield a group response (arrow 4). This response is then implemented or transferred to external verification (arrow 5).

Lastly, many studies have reported that positive emotions increase team creativity (for reviews, see (Paulus and Dzindolet 2008) and (Shin 2014)). This can be explained with the broaden-and-build theory (Fredrickson 1998), according to which positive emotions have the ability to broaden an individual’s momentary thought–action repertoire, which in turn promotes the discovery of creative ideas and actions.

### Serious games

Games have been a part of human civilization for thousands of years (Akarsu et al. 2018) and have helped in making daily life and reality more interesting and engaging (McGonigal 2011). Based on a story by Herodotus (440 BCE), 3 millennia before his time, the Lydians invented several games, including dice, knuckle-bones, and the ball, to overcome an 18-year-long famine (Dal Sasso et al. 2017). “The plan adopted against the famine was to engage in games for one day so completely as not to feel any craving for food […] and the next day to eat and abstain from games” (McGonigal 2011). That game-based remedy can be explained by flow theory (Csikszentmihalyi 1991). According to Csikszentmihalyi (1991), playing games is one of the activities that helps participants achieve a state of mind which is characterized by complete absorption in what they are doing, and a resulting altered sense of time. This mental state in which a person performing an activity is completely immersed in a feeling of energized focus, full engagement, and enjoyment in the process of the activity, is called flow (Csikszentmihalyi 1991). Later on, Sawyer (2007) expanded flow theory and argued that a group can enter a state of flow to increase their innovation.

A typical game is as a combination of a game system (e.g., game board) with which participants interact, rules of interactions, and a goal (Yilmaz 2013; Dal Sasso et al. 2017).

The famous book by Neumann & Morgenstern (1944) titled “Theory of games and economic behavior” is the cornerstone upon which modern-day game theory is based. According to game theory, games fall into three broad categories (Zagal et al. 2006; Daylamani-Zad et al. 2020):

- *competitive games*, where individuals form strategies that directly oppose to others;
- *cooperative games*, where players have interests that are neither completely opposed nor completely coincident; hence, opportunities exist for cooperation but not necessarily for equal reward;
- *collaborative games*, where all players work together as a team, sharing a common goal, outcomes, and penalties.

Driven by the successes of the leisure game industry, the notion of games was redefined in non-gaming application areas, including learning, coaching, health care, awareness raising, strategic thinking, as well as market research (Gelperin 2011; Pallot et al. 2012; Yilmaz et al. 2016; Lamrani et al. 2017; Westera 2017; Stettina et al. 2018; Trujillo et al. 2018; Üsfekes et al. 2019; Lamrani and Abdelwahed 2020; Butt et al. 2021a; Khorram et al. 2021). Abt (1970) coined the term “serious games” to describe games which “are not intended to be played primarily for amusement,” but are designed for educating or solving a problem.

### Retrospective games

Retrospective games are a form of collaborative games used for agile retrospectives. They refer to several structured social activities inspired by game play and designed to help the participants share their knowledge and experiences on a given topic, explore this knowledge in ways that may not occur during normal interactions, identify hidden assumptions, develop a joint understanding of a problem, and generate innovative ideas about solving the problem (IIBA, 2015; Gonçalves and Linders 2014; Przybylek and Olszewski 2016; Yilmaz and O’Connor 2016; Matthies 2020; Matthies et al. 2020). They involve a strong visual or tactile element (IIBA, 2015). Activities like moving sticky notes, scribbling on white boards, assembling things, or drawing pictures encourage less vocal people to contribute to the discussion, help overcome inhibitions, and foster creative thinking (Hohmann 2006; Lin et al. 2010) by stimulating alternative mental processes. They have also been claimed to help agile teams get better outcomes from retrospective meetings (Roden and Williams 2015).

Table 1 briefly presents the retrospective games that we used in our prior study. All of them use a game board, but different metaphors and categories are employed. The facilitator starts each game by drawing its board and distributing several packets of sticky notes in as many colors as the number of categories in the game.

**Table 1:** Retrospective games

Game	Game board	Categories
Sailboat	an image of a sailboat, rocks, wind, an island	Island – the team’s goal; Anchor – everything that slows the team down; Rocks – the risks the team might encounter; Wind – everything that helps the team reach their goal
Mad/Sad/Glad	three columns	Mad – frustrations, issues that have annoyed the team and/or have wasted a lot of time; Sad – disappointments, issues that have not worked out as well as was hoped; Glad – pleasures, issues that have made the team happy
Mood++	five columns	* – all categories from Mad/Sad/Glad; Flowers – appreciation to teammates who have done something magnificent for the team or a particular team member; Ideas – suggestions how to improve the teamwork or the process
Starfish	an image of a starfish with five arms	Stop Doing – activities or practices that do not add value, or even worse, are hindrances to progress; Less Of – activities or practices that have been done and have added value but have required more effort than really needed; Keep Doing – activities or practices that the team is doing well and wants to keep; More Of – activities or practices that are useful but not fully taken advantage of; the team believes that they will bring more value if they are done even more; Start Doing – activities or practices that the team believes will bring value and will improve current processes
5Ls	five columns	Liked – what did the team really appreciate about the Sprint? Learned – what new things has the team learned during the Sprint? Lacked – what things could the team have done better in the Sprint? Longed For – what things did the team wish for but were not present during the Sprint? Loathed – what things did the team dislike in the Sprint?

The **Sailboat** game (Gonçalves and Linders 2014) uses the metaphor of a sailboat, rocks, clouds, and islands. The sailboat stands for the team. Everyone wants the sailboat to move fast to reach the island. Unfortunately, the boat has a few anchors holding it back. The game motivates team members to be focused on future directions, where they want to go. It also helps the team to identify impediments, possible risks, and things that make them deliver great software.

The **Mad/Sad/Glad** game (Derby and Larsen 2006) helps release a heavy emotional steam and connect team members’ emotions to events that happened in the Sprint. The emotions are often affected by problems encountered while working together. Knowing these problems can help the team solve future problems. **Mood++** extends Mad/Sad/Glad by introducing two new categories (Przybyłek and Kotecka 2017).

Both the **Starfish** (Gonçalves and Linders 2014) and **5Ls** (Przybyłek and Kotecka 2017) games are an evolution of the typical three questions that are used for retrospectives. In contrast to Mad/Sad/Glad, they stimulate the team to think mostly from a rational perspective, rather than an emotional perspective.

There are also non-board retrospective games. One of the most prominent among them is the 360 Degrees Appreciation (Caroli and Caetano 2016), which fosters open appreciation feedback within a team. The game provides an opportunity to acknowledge teammates’ hard work, contribution, and help. It is especially useful after a tough iteration to raise team morale. It consists of the following steps:

- each team member writes down on a sheet of paper what he/she appreciates;
- team members form a circle;
- one participant sits in the center of the “circle of recognition”;
- everyone in the circle reads the appreciation feedback to the team member in the center (complete the 360 degrees);
- team members change in the center until everyone has received feedback.

## Related work

The application of collaborative games in software development is receiving more and more attention. An important cornerstone for this research area was the games introduced by Hohmann (2006) as market and product research techniques and later adopted by Ghanbari et al. (2015) and Przybyłek and Zakrzewski (2018) to enhance requirements engineering processes. Being inspired by Hohmann’s games, Trujillo et al. (2014) proposed a game-based workshop as an alternative for the Inception phase of a project. Besides this, Gelperin (2011) defined six collaborative games to support requirements understanding and proposed a mapping system to help developers choose the best game to play in any situation. Our current work differs from those cited above in that we use collaborative games to boost retrospective meetings, while they focused on games that stimulate collaboration and communication between the development team and its stakeholders.

In addition, considerable research has been directed at adopting, specifying, and promoting collaborative games to facilitate agile retrospectives. Lamoreux (2005) reported that in their organization, the retrospective was one of the most challenging agile practices to implement and they encountered many roadblocks to effective reflection. Their initial reluctance to have regular retrospectives was overcome after they adopted the Conversation Café technique, which can be considered to be a retrospective game. Derby and Larsen (2006) presented a general agenda for retrospective meetings, i.e., set the stage, gather data, generate insights, decide what to do, and close the retrospective, and proposed games for each phase. Their work was continued by Gonçalves and Linders (2014), Krivitsky (2015), Roden and Williams (2015), Caroli and Caetano (2016), and Baldauf (2018) who described, respectively, 13, 16, 50, 44 and 130 retrospective games. Krivitsky (2015) also provided the details of the games based on the team mood, size, and proximity. Jovanović et al. (2016) then proposed a new classification of retrospective games based on the four-stage group development model by Tuckman and cataloged 89 games. More recently, their research was extended by Mesquida et al. (2019) who proposed two classification systems based on team maturity and the stage of the meeting. They also created a ready-to-use toolbox of 12 retrospective games with the main objective of improving communication, cohesion, and coordination. In addition, Marshburn (2018) proposed an experimental design to be used in future research to evaluate the effectiveness of game-based retrospectives vs. non-game-based retrospectives in a controlled experiment.

However, none of the above work has evaluated how retrospective games work in practice. To the best of our knowledge, our prior pilot study (Przybyłek and Kotecka 2017) was the first to investigate the impact of retrospective games on social factors such as participants’ communication, motivation-and-involvement, and creativity. Recently, our pilot study has been replicated by Ng and her team (Wawryk and Ng 2019; Ng et al. 2020; Mich and Ng 2020) in Bluebay Poland, IHS Markit Gdańsk, and Intel. They have confirmed our initial findings that game-based retrospectives produce better results than standard retrospectives and develop teamwork qualities. Nevertheless, they have not strengthened the research rigor. Encouraged by the preliminary results, in this paper, we explore retrospective games further in a systematic way.

Finally, Kua (2013) identified common retrospective problems, while Matthies et al. (2019) connected the problems to specific retrospective games that may address them. In a subsequent study (Matthies and Dobrigkeit 2020), the proposed mapping was evaluated by student as well as industry teams. The obtained results suggest that retrospective games address retrospective problems in accordance with the mapping. The retrospective problems identified in the aforementioned work were used in the Diagnosing phase of our current study when preparing the interview protocol. Besides, our results confirm that game-based retrospectives at least mitigate common retrospective problems.

## Study design

In this section, we: (1) describe the research method we adopted to conduct our study; (2) define the research questions; (3) present the research context; and (4) discuss the data collection and analysis procedures.

### Research method

The study was conducted as Action Research (AR) since this method is well suited for studying new techniques or approaches in practitioner environments (Baskerville 1999; Staron 2020). In AR, researchers intervene in the studied situation for the explicit purpose of improving the situation (Hult and Lennung 1980; Easterbrook et al. 2007). AR involves dual relevance objectives – conducting research relevant to the participant organization while expanding scientific knowledge (Baskerville and Myers 2004). Accordingly, it synergistically and holistically associates research and practice so that research informs practice and vice versa (Avison et al. 2017).

Compared to controlled experiments, AR is focused on doing research with and for people, rather than experimenting on them (Reason and Rowan 1981; Staron 2020). It also differs from case studies, where the researcher only observes and comments on organizational phenomena but does not change them (Yin 2018). On the contrary, the action researcher is concerned with planning and creating organizational change and then reflecting on the results (Baburoglu and Ravn 1992).

AR can be initiated either by practitioners or by researchers (Root-Bernstein 1989). The former case is problem-driven initiation, in that practitioners might be confronted by a seemingly intractable problem and looking for help from academics (Avison et al. 2001). In this situation, the researchers have an opportunity to develop their research program somewhat opportunistically, undertaking a series of research projects that have a broad theoretical span (Avison et al. 2007). The latter case is research-driven initiation, in that the action researchers might be in possession of ideas or general theoretical approaches to addressing problem situations and searching for settings that are characterized by such problems to apply and test their concepts in a real-world scenario (Avison et al. 2001; Nguyen and Swatman 2003). In this situation, the practitioners may be somewhat doubtful or indifferent, so the researchers must bring forward a convincing vision of the practical benefits for the host organization. In either case, the researchers bring their knowledge of AR and general theories, while the practitioners bring their practical knowledge and context (Baskerville and Myers 2004).

**Fig. 2:**
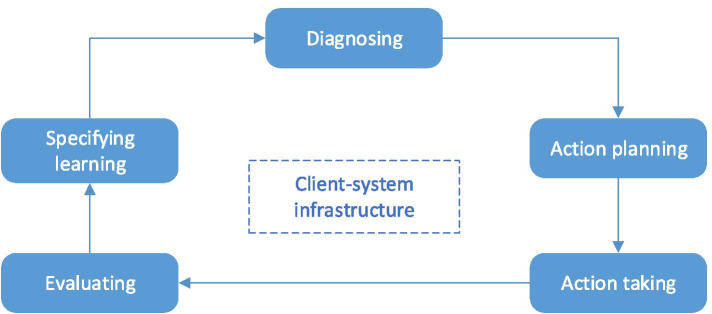
The Canonical Action Research cycle (Susman 1983)

Among the multiple forms of AR (for review, see (Baskerville and Wood-Harper 1998)), the most popular in the information systems domain is Canonical Action Research (Baskerville and Wood-Harper 1998; Baskerville 1999; Davison et al. 2004; Marcinkowski and Gawin 2019). It was originally proposed by Susman and Evered (1978) and later revised by Susman (1983) as a cyclical process consisting of five interdependent phases (Fig. 2):

- **Diagnosing**. The current organizational situation is diagnosed in order to identify the primary problems that are the underlying reasons for change (Baskerville 1999).
- **Action planning**. The researchers together with practitioners plan actions to address the identified problems.
- **Action taking**. The planned intervention is implemented.
- **Evaluating**. The actors determine whether the expected effects of the intervention were achieved, and whether these effects remedied the problems (Baskerville and Wood-Harper 1998).
- **Specifying learning**. The actors reflect on the intervention and its effects and document findings that will add to the body of knowledge of the discipline. They also reach a decision whether to finish the project or to proceed through an additional process cycle (Davison et al. 2004).

Canonical Action Research is carried out as one or several cycles of the above activities (Baskerville and Wood-Harper 1998; Davison et al. 2004). Further to this, Davison et al. (2004) defined five principles (Table 2) that researchers and reviewers can use both to ensure and to assess the rigor and relevance of canonical action research.

**Table 2:** Principles of Canonical Action Research

**The Principle of the Researcher–Client Agreement (RCA)**	
It provides a guiding foundation for an AR project. By specifying the scope and objectives of the project as well as the co-operation between the researcher and client and involving key stakeholders in its creation, the agreement constitutes the research environment and promotes a spirit of shared inquiry.	
**The Principle of the Cyclical Process Model (CPM)**	
It requires the project to be conducted in a precise and sequential manner according to the five phases model by Susman and Evered (1978).	
**The Principle of Theory**	
It emphasizes the importance of applying one or more theories to guide the project activities and relate the findings to existing theories (Davison et al. 2004).	
**The Principle of Change through Action**	
It reflects the essence of Action Research, which is to implement intervention in order to change the current situation and its unsatisfactory conditions.	
**The Principle of Learning through Reflection**	
It guarantees that both researchers and practitioners draw insights from what they have learned and identify implications for other situations and contexts.	

A severe limitation of AR is the restriction to a single organization, which hinders generalization of the results (Nguyen and Swatman 2003; Sjøberg et al. 2007a). Therefore, to test whether the approach from our previous AR project (Przybyłek and Kotecka 2017) can be repeated with similar results in other contexts and settings, we decided to search for several host organizations as sites for a new study. We adopted the joint replication approach (Krein et al. 2016) proposed in the context of a controlled experiment. Joint replication is “a multi-site study, performed by multiple research teams whose efforts are coordinated, yet the researchers at each site act independently in performing their own replication” (Krein et al. 2016). The research teams still explicitly communicate about important aspects of the study, including adopting common interview protocols, questionnaires, as well as an interventions. Nevertheless, each team carries out the intervention, collects data, and performs the initial data analysis separately, after which the data is then merged and analyzed together. In analogy to the multiple case study design, we call the resulting design a multiple Action Research design. Consistent findings from multiple organizations will not only be viewed as more compelling, but will allow us to develop a middle-range theory (Wieringa and Daneva 2015). Moreover, the new research design will mitigate the inherent bias of the approach due to the researchers’ subjective point of view.

Considering that a lot of software development companies had previously collaborated with our university, we opened a call for a new AR project. We promoted our project as an opportunity for a company to improve its work practices related to the Sprint Retrospective and enhance its teams’ competencies. We also brought into play both image (i.e. companies may find it attractive to be named as research partners in research publications) and altruistic (i.e. the study results will not only help the organization, but also the rest of the world) benefits (Prechelt et al. 2015). According to Root-Bernstein (1989), this kind of genesis of an AR project is referred to as research-driven initiation. Several companies’ representatives applied for our call and they were interviewed to make sure that their teams were experiencing retrospective problems and they were willing to engage in an effort to overcome them. At the end of the day, OKE Poland, Dynatrace, and SentiOne were chosen as the host organizations, while their representatives (Wojciech Kowalski, Marta Albecka, and Olga Springer, respectively) became co-researchers as well as co-authors of the study. Thereby, according to the maturity model by Wohlin (2013), we reached the highest level of closeness between industry and academia.

Table 3 presents the positions of the managers who sanctioned our research project in each host company. Before approving the participation of their teams in the research, the team leaders discussed the idea with the team members and explained how the project might benefit them. As the idea was warmly welcomed, implicit consent was presumed from a failure to dissent. Nevertheless, it is not clear whether individuals had the right to refuse to participate in the research. Since the intervention was carried out at the team level rather than at the individual level, either the entire team could participate or none could. Still, team members could have refused to complete the survey or participate in the focus group and they were informed that the collected data might be published.

**Table 3:** Companies’ consent to carry out the research at different levels of organizational hierarchy

	OKE Poland	Dynatrace	SentiOne
top-level management	CEO		CTO, COO
middle-level management		development lead	
low-level management	team leader	team leader	team leader

Since AR creates organizational change, we followed Lenberg et al.’s directions (Lenberg et al. 2017) for increasing software engineers’ support for change initiatives and, thus, to increase the likelihood of a successful change. Lenberg et al. (2017) identified three underlying concepts with an expected significant impact on software engineers’ attitudes toward organizational change, i.e., their knowledge about the outcome of the planned change, their understanding of the need for change, and their feelings of participation in the change process. Insights on the expected benefits of game-based retrospectives were provided to the participants based on our experience from the pilot study. As for the second concept, the reasons behind the initiation of the change were discovered by the participants themselves (see Table 6). In turn, the active participation of the employees in the change process is a crucial characteristic of AR (Baskerville and Myers 2004; Staron 2020).

### Research questions

To guide our study, we defined the following research questions:

1. How do game-based retrospectives impact common retrospective problems?
2. How do retrospective games impact teamwork qualities?
3. What are the advantages and disadvantages of the adopted games?
4. What are the lessons learned in adopting retrospective games?

### Research context

Describing the research context is of importance as it allows the conditions under which the results were produced to be understood (Petersen and Wohlin 2009). Our project was a collaborative study between Gdańsk University of Technology and three software development companies. We followed Susman’s AR design (Susman 1983) and conducted one complete cycle of AR. The research in each host organization took between 4 to 8 months, and the project was carried out by three two-person research teams. Each team was dedicated to one company and consisted of the company representative (an internal researcher) and the leader of the whole project (an external researcher) who also coordinated all teams. Two Scrum teams from each company participated in the project. Since AR requires a breadth of pre-understanding of the organizational environment (Coghlan and Brannick 2005), each host organization and the participating teams are described in the subsequent subsections, while the compositions of the teams are summarized in Table 4.

**Table 4:** Participating teams and their compositions (SM denotes Scrum Master, while PO denotes Product Owner)

OKE_A	1x Lead Programmer & SM	OKE_B	1x Lead Programmer & SM & Proxy PO
	2x Junior Programmers		1x Senior Programmer
	1x Tester & PO		3x Programmers
			1x Tester
Dyna_A	1x Team leader & PO & SM	Dyna_B	1x Lead Developer & SM & PO
	3x Senior Developers		1x Senior Developer
	4x Developers		3x Developers
	2x Junior Developers		
Senti_A	1x PO	Senti_B	1x PO
	1x Quality Manager		1x Project Coordinator
	1x Product Designer		4x Senior Research Engineers
	2x Senior Developers		1x Junior Research Engineer
	3x Developers		1x Linguist
	1x Tester		

#### OKE Poland (https://oke.pl)

OKE Poland was established in association with international partners to create user-friendly IT solutions. Currently, the company is mainly focused on improvement and development in the hybrid TV platforms area.

**Team A** (OKE_A) implements a business-to-business platform that allows companies to outsource and hire people in order to maintain a flexible workforce. Most of the implementation tasks are carried out by 2 junior programmers. The team also comprises a tester and a lead programmer who also plays the role of Scrum Master. Since the tester is the originator of the platform, he has also become the product owner.

**Team B** (OKE_B) develops and maintains a complex system which offers customer management, service integration and management, and content management. Their client is M7 Group, which is a pay-tv operator offering language-specific packages to over three million subscribers in eight European countries. M7 provides its customers with a wide variety of programs that can be watched at any time, any place, and via any screen. Team B consists of 2 senior programmers with 10+ years of experience, 3 programmers, and 1 tester. The most experience programmer also leads the team and fulfills the role of Scrum Master and Proxy Product Owner. He communicates with the Product Owner regularly during the sprint. The whole team travels to the Netherlands a few times per year to meet with the Product Owner and business stakeholders.

#### Dynatrace (https://jobs.dynatrace.pl)

Dynatrace builds intelligent software designed to help companies manage their application performance and understand how the application performance impacts the users. The delivered tools allow DevOps and admins to leverage full stack monitoring by collecting data from across all components of a modern application and infrastructure stack.

**Team A** (Dyna_A) maintains and evolves Dynatrace Managed, which is the on-premise deployment model offered by the Dynatrace monitoring tool. The team is responsible for maintaining the web page that presents the accounts and licenses as well as providing proper activation emails and installation options. The team consists of 9 developers with experience ranging from 2 to 20 years, who are led by the Product Owner and Scrum Master in one person. Before becoming the team leader, this person was also a developer in the team.

**Team B** (Dyna_B) develops a custom sign-in page for all elements of the Dynatrace product. Their product is still in the development stage, so they can work at their pace, because, in contrast to Team A, they are not bothered by bugs reported by the customers. The team includes 5 developers. Each developer is responsible for the whole product. There are no divisions for front-end programmers, back-end programmers, and testers. There are two senior developers, whereas the remaining team members have no more than 5 years experience. The most experienced developer also leads the team and plays the role of Scrum Master and Product Owner.

#### SentiOne (https://sentione.com/)

SentiOne builds a Conversational AI Platform – product providing state-of-the-art customer service automation based on social listening and data analytics.

**Team A** (Senti_A) maintains and develops the front-end and back-end of SentiOne’s applications, viz. Listen, React and Automate. The team consists of 5 software developers with experience ranging from 3 to 10 years, one Quality Manager, and one Software Tester. The team has a dedicated Product Owner and Product Designer, which are representatives of the Product Team. There was no Scrum Master.

**Team B** (Senti_B) works on a grant project. The main result of their work is a natural language understanding engine. They make experiments and try different approaches to deliver the best results every sprint. They work closely with Linguistics to prepare datasets and test outcomes. The team consists of 5 Research Engineers whose experiences range from 0.5 years to 7 years, a Project Coordinator (specialized in NLP), a Linguist representative, and a Product Owner. There was no Scrum Master.

### Data collection and analysis

As recommended by DeLuca et al. (2008) and Easterbrook et al. (2007), we used several data collection and data analysis techniques to reduce the overall risk to validity and to increase the overall rigor of the study. During the diagnosing phase, the data originated from interviewing. Then, during the action-taking phase, the internal researchers conducted “complete participant observations” (i.e. the researcher was totally involved as a participant) according to Spradley’s taxonomy (Spradley 1980). According to Seaman (1999), software developers reveal their thought processes most naturally when communicating with other colleagues, so this communication offers the best opportunity for a researcher to observe the software development process. Therefore, the internal researchers wrote field notes on the observations on how participants interacted. Unfortunately, during the review process of this paper, it turned out that they had missed out writing down some important facts (e.g. specific examples of usefulness) and when revising the paper, they had to resort to their memories.

Finally, we used both questionnaires and focus group sessions (Kontio et al. 2008) for evaluation of our approach to form a fuller picture from a wider range of coverage (Kaplan and Duchon 1988). While questionnaire results revealed team members’ perceptions of retrospective games, focus group discussions allowed in-depth exploration of the reasons why the participants thought the way they did. From a variety of forms of focus group meetings (Staron 2020), we chose “moderated discussion” as it is suitable for generating a common understanding or common view on a topic. In this form, participants discuss a set of predefined ideas and issues while the facilitator assures that everyone has an opportunity to participate, and the discussion stayed focused (Staron 2020). A brief summary of the data collection techniques used at each phase of our AR project is summarized in Table 5.

**Table 5:** Data collection techniques (Note that we did not collect data during action planning and specifying learning)

AR phase	Data collection technique	Data source	Objective
Diagnosing	unstructured interview	team leaders and/or scrum masters	To find the adoption of Scrum practices in the participating teams.
	semi-structured interview	team members	To identify retrospective problems in the participating teams.
Action taking	participant observation	researchers	To take a close look at how the game-based retrospectives proceed.
Evaluating	questionnaire	team members	To assess the adopted games.
	focus group	team members	To discuss the results of the intervention and collect “lessons learned” recommendations.

As for the questionnaire, the attitudes of the participants were assessed using individual Likert-type items (Clason and Dormody 1994). For each statement, there were five choices: Strongly Disagree (1), Somewhat Disagree (2), Neither Agree nor Disagree (3), Somewhat Agree (4), and Strongly Agree (5) accordingly. We utilized diverging stacked bar charts as well as spider charts to analyze the Likert-type items.

We used content analysis to analyze and interpret the data from the focus group sessions. Content analysis is a formal type of qualitative method for “subjective interpretation of the content of text data through the systematic classification process of coding and identifying themes or patterns” (Hsieh and Shannon 2005; Defranco and Laplante 2017). Content analysis may be used in either an inductive or deductive way depending on the purpose of the study (Elo and Kyngäs 2008). The fundamental difference between the two approaches centers on how the initial codes or categories are developed (Cho and Lee 2014). Inductive content analysis is used when former knowledge regarding the phenomenon under investigation is limited or fragmented (Elo and Kyngäs 2008). In this approach, codes, categories, or themes are derived from the data. In contrast, deductive content analysis is used when the structure of analysis is operationalized on the basis of previous knowledge understood about the topic (Elo and Kyngäs 2008; Defranco and Laplante 2017). Accordingly, the deductive approach starts with predetermined codes, categories, or themes derived from prior relevant theory, research, or literature (Cho and Lee 2014). In our study, we utilized the deductive approach following Mayring’s procedure (Mayring 2014) that consists of:

- research question,
- theoretical-based definitions of categories,
- theoretical-based formulation of coding rules,
- revision of categories,
- final working through the text, and
- interpretation of the results.

## Action research cycle

This section reports on all five phases of the conducted Action Research cycle, i.e. diagnosing, action planning, action taking, evaluating, and specifying learning.

### Diagnosing

A prerequisite to determine an appropriate intervention is a detailed understanding of not only the problematic situations but also the surrounding environment (Davison et al. 2004). Thereby, we started with an investigation into the current adoption of Scrum practices. Since each internal researcher was a member of one of the participating teams, we already had information about 3 teams. Consequently, we conducted unstructured interviews with the team leaders, scrum masters and developers of the remaining teams. Table 6 lists the collected information. From this table, we can see that except for Dyna_A, the teams did not used Scrum as it is intended to be used. The implementation of new user stories to satisfy customer needs had a higher priority than following agile practices. Besides this, OKE_B, Dyna_A, and Senti_B worked under time pressure as well as top-down changed targets and priorities. Although all host companies had adopted agile methods, their transformation to implement agility at an organizational level was still underway. As bureaucratic organizations, they relied on a traditional hierarchical and command-and-control management and did not involve the teams when making strategic project decisions, which contradicts self-managing teams (Nerur et al. 2005; Moe et al. 2012). Overall, our findings suggest that neither organizational culture nor managers’ mindsets can be easily changed.

**Table 6:** Adoption of Scrum in the participating teams

Team; # team members	Scrum compliance	Sprint length; development / maintenance
OKE_A; 4	No Daily Scrum due to the following reasons: • team members usually have only a few hours of overlapping time during the workday, • team members are distributed among different rooms in the office space, • the tester and the team leader are also engaged in other projects. Sprint Planning is held at the beginning of each sprint. Sprint Review and Sprint Retrospective are merged into one meeting, which usually takes place on a regular basis at the end of each sprint.	1 week; 40% / 60%
OKE_B; 6	Daily Scrum is held every day. Sprint Planning is done by the Product Owner and the team leader, while the results are communicated to the developers. Sprint Review meeting is replaced by a Sprint Review report in which every developer describes the functionality that he/she has implemented in the sprint. The report is sent to the Product Owner. Sprint Retrospective is usually skipped, because the team constantly works under time pressure to deliver the increment.	flexible, 1 week during the research; 50% / 50%
Dyna_A; 10	Strictly adhere to Scrum, but they are forced to do it. Sometimes a simplified version of Mad/Sad/Glad or Starfish has been used during the Sprint Retrospective, but only a few team members consider this approach useful. We traced the underlying cause of the problem to the replacement of sticky notes for writing directly on the whiteboard, which leads to the situation where only part of the team is involved in writing. Besides, almost half of the team members would not attend retrospectives if their attendance was not obligatory.	2 weeks; 40% / 60%
Dyna_B; 5	Daily Scrum, Sprint Planning and Sprint Review are conducted in accordance with the Scrum Guide. Sprint Retrospective is done at times and there is no specific time for it. The meetings are considered rather boring, because they do not go beyond the typical three retrospective questions. Nevertheless, only one team member would skip the meeting if it was not obligatory.	2 weeks; 65% / 35%
Senti_A; 9	Daily Scrum, Sprint Planning and Sprint Retrospective are conducted in accordance with the Scrum Guide. Moreover, all team members willingly participate in retrospectives. Sprint Review is replaced by a Demo meeting, which is organized every quarter by the Product Team to update the stakeholders about recent key changes and to plan for the next months. In addition, Backlog Refinement is organized every sprint to estimate the cards in the Product Backlog.	2 weeks; 70% / 30%
Senti_B; 8	Daily Scrum, Sprint Planning and Sprint Review are conducted in accordance with the Scrum Guide. Sprint Retrospective is held very rarely, because the team works constantly under the pressure of grant deadlines. In addition, Backlog Refinement is organized every sprint to estimate the cards in the Product Backlog. All meetings are added to the calendar, however they are rescheduled very often.	2 weeks; 90% / 10%

Next, we focused on diagnosing the retrospective meetings. We carried out a series of face-to-face semi-structured interviews across the team members. The interview protocol was based on common retrospective problems identified by Kua (2013) and Matthies et al. (2019) and was structured around the following questions:

- Do your retrospectives bring added value or are they a waste of time?
- Are your retrospectives too repetitive?
- Are your retrospectives thoroughly prepared and well structured?
- Do all participants contribute to the discussion or it is dominated by a few vocal people?
- Is there a tendency to view your retrospectives as a chance to complain instead of a chance to improve?
- Would you attend retrospectives if your attendance was not obligatory?

We chose interviews over a focus group because we were asking about sensitive issues, so it would be uncomfortable answering honestly in front of other teammates (especially the Scrum Master). Besides, the interviewees were guaranteed that sensitive information would not be possible to trace back to individuals. Note that members of Senti_B were not able to answer the questions, because the vast majority of them were new to Scrum and had hardly any experience in retrospectives.

Table 7 summarizes the collected information. Although the participating teams varied according to their adoption of Scrum, all of them encountered most of the common retrospective problems. Nevertheless, even though the problems occurred, they were not necessarily severe. For instance, some Senti_A members pointed out that neither repetitiveness nor “too many complaints” was a big issue. Moreover, as a consequence of these problems, the interviewers raised several other issues, such as:

- the same ideas come up every time;
- there is not enough to talk about based on a single Sprint;
- there is no good discussion on how to improve the teamwork;
- wasting time on long, ineffectual debates on one issue while neglecting other issues due to a lack of time;
- difficulties in writing down ideas expressed in long unstructured oral statements;
- retrospectives are too short to come up with solutions to complex problems, so additional meetings should be planned;
- it is hard to prioritize issues and focus on the most important ones, because team members want to discuss and explore potential solutions for all of the problems in one meeting.

**Table 7:** Retrospective problems in the participating teams

Retrospective problem	OKE_A	OKE_B	Dyna_A	Dyna_B	Senti_A	Senti_B
little added value	++	+	+++	++	+	n/a
too repetitive	+++	++	+	+++	+++	n/a
lack of structure / preparation	+	+		+	+++	n/a
unequal participation	++	+++	++	+		n/a
too many complaints		+	++	+	++	n/a

Frequency of problem occurrence:

+ fewer than 1/3 of team members have noticed the problem (but at least one)

++ between 1/3 and 2/3 of team members have noticed the problem

+++ more than 2/3 of team members have noticed the problem

Note that most of the encountered difficulties had been introduced through inadequate practices or resulted from an ad hoc approach. Brooks (1987) refers to them as “accidental difficulties” and claims that they can be addressed by a disciplined software engineering process. He also argues that the great leaps of progress in the past were accomplished by eliminating accidental difficulties. In this research, we planned to adopt retrospective games as a systematic approach to conducting retrospective meetings.

### Action planning

The diagnosis phase provided valuable insights into the current situation. The collected information confirmed that our original research problem was authentic, which is a prerequisite for AR, and that the participating teams were appropriate to implement retrospective games. Initially, we suggested implementing games that we had successfully adopted in our previous study (Przybyłek and Kotecka 2017) except for Mad/Sad/Glad, which seemed to be superseded by Mood++. All of these games were ranked as the most popular retrospective activities (Dzieciątek 2019). We wanted each game to be implemented twice in each team, but we did not suggest the order in which the games would be introduced, leaving this decision to the meeting facilitators.

**Fig. 3:**
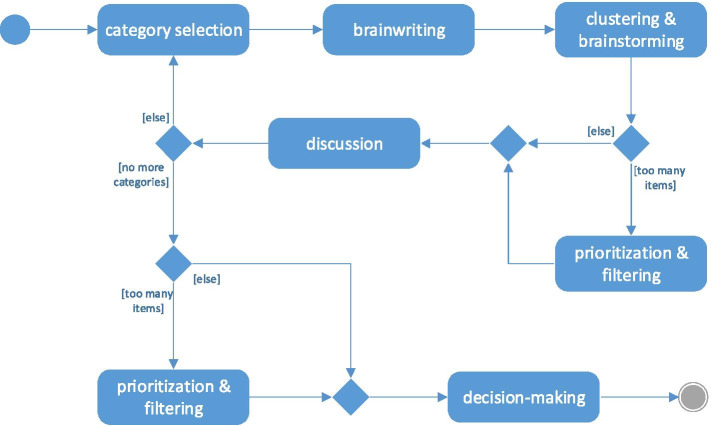
UML Activity Diagram representing the flow of the collaborative game process

Drawing on Paulus and Nijstad’s (2003) group creativity model as well as the state-of-the-art in group idea generation, we proposed a generic model of running a game-board collaborative game. The model shows how to orchestrate the flow of information and ideas (Fig. 3). The rationale and motivations behind our approach are as follows.

Firstly, we followed Osborn’s (1957) suggestion that breaking a problem down into different components makes idea generation more effective. Accordingly, each category in a game is processed separately. The order in which to process the categories is indicated by the facilitator, but usually she/he should adhere to the order in which they are listed in Table 1.

Secondly, we wanted to have individuals participate in both individual and group brainstorming sessions. Following the findings of Baruah and Paulus (2008), we decided that individuals first generate their ideas in an unconstrained fashion by brainwriting, and then proceed to brainstorming, where exposure to the ideas of others can stimulate additional ideas due to associative processes (Korde and Paulus 2017). In brainwriting, each team member silently writes on separate sticky notes his/her ideas, feelings, or feedback that could be assigned to the processed category. It is important to have one idea per sticky note (sticky notes will be clustered into themes). After a set amount of time, the participants post their notes. Then, the facilitator reads each note aloud so that everyone can become aware of it. The author can briefly explain the purpose of the note to assure a common understanding. If anyone’s idea inspires others to write more, new sticky notes may be posted. The facilitator, with the help of the team, groups related ideas into logical themes as they are presented. Next, the themes are discussed one-by-one and corrective actions are proposed. Finally, participants reach a consensus and develop an action plan to be implemented for the next iteration.

Thirdly, we introduced an optional activity, i.e., prioritization & filtering. If there are too many items to proceed, participants use dot-voting to prioritize and converge on the most important ones. It is crucial to limit the number of items, because having too many alternatives can inhibit one’s search of the full range of possible aspects of a problem (Chua and Iyengar 2008). Besides, when participants are considering a broad ranging problem, they tend to narrow their focus in order to gain consensus (Baruah and Paulus 2008). Each participant is entitled to the same number of votes and she/he may place any number of her/his dots on any item. Since people tend to conform to the majority view, even when they know it is wrong (Asch 1956), participants should cast their votes all at once. When the voting is over, the facilitator sorts the items based on the number of votes each received.

Furthermore, we prepared a questionnaire to capture the perception of each game and its effects. The questionnaire comprised seven Likert-type questions (see Appendix 1). The evaluation of all games would be done using one questionnaire sheet to make it easier to assess each game against the others. We planned to distribute the questionnaire sheets just after the meeting is over and then collect them back and store them until the next Retrospective. The team members would be allowed to change their assessments of previous games when assessing a new game. Finally, we planned to conduct a focus group with each team to gather further insights about their perceptions, opinions, and attitudes toward game-based retrospectives. The discussion would be structured around six predefined questions (see Appendix 2).

**Fig. 4:**
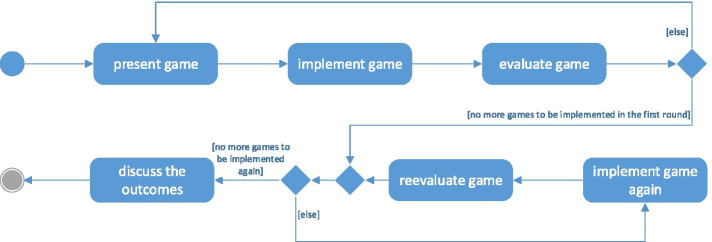
UML Activity Diagram representing the planned actions

We arranged a series of meetings with the Scrum Masters and team leaders to present and discuss our proposal. We were open to changes in the agenda, but our condition was to agree with all of the teams on the same set of games and questions for the questionnaire and focus groups. During the discussion, it turned out that some teams that worked under considerable time pressure not only were not able to conduct retrospective after each sprint, but also to spent time on learning the rules of the game at the beginning of each meeting. Thus, it would take too much time to implement all of the games twice in these teams. Accordingly, we agreed that it would be up to the facilitator to decide whether to implement a game twice. If the facilitator deems that a game has not demonstrated its full potential due to a bland sprint, she/he will introduce it again. Someone also suggested introducing one more game, namely 360 degrees appreciation, and the proposal was approved. OKE_A also agreed to split the Review&Retrospective meeting into two separate meetings as specified in the Scrum Guide (Schwaber and Sutherland 2020), while OKE_B agreed to introduce the Sprint Retrospective at the end of each sprint. The outline of the common intervention into all participating teams is illustrated in Fig. 4.

### Action taking

With the help of the Scrum Masters, we started to implement the intervention as specified in the action planning phase. Some team members were so excited about retrospective games that they asked for the documentation on the next game to prepare ahead for the meeting. Nevertheless, even though all team members had been familiarized with the documentation, we still explained the game, since as shown by Robey et al. (2002) effective training strengthens employees’ commitment to change.

After the second game was deployed in OKE_A, we came across the first problem – two programmers left and were replaced by new ones. Consequently, the team decided to discard the results collected so far and to introduce both games again. Moreover, OKE_A and OKE_B committed themselves to implementing retrospective games in all subsequent retrospective meetings in order to reduce the risk of a similar situation happening again. Thereby, both OKE teams completed the research process first and we even conducted a focus group meeting with them to discuss the results when the process was still in progress in the other teams. Since both OKE teams claimed that Mood++ took too much time when compared to other games, we asked the other teams to also introduce the Mad/Sad/Glad game, which is a simpler version of Mood++. Later on, it turned out that employee turnover was also a problem in Dyna_B (one developer left during the research) and Senti_B.

Another problem faced mainly by big teams was sickness absence or holidays. Some teams mitigated this problem by playing most of the games twice, while others tried to either postpone a game when someone was absent or repeat a particular game. Besides this, in Senti_B, one team member, who works remotely full-time, attended the meetings via Google Hangouts. The same approach was used occasionally in both SentiOne teams when someone worked from home that day. The teams believed that this approach did not affect the quality of the meeting.

Finally, the Dyna_A Scrum Master decided not to complete the questionnaire, because he could be biased.

### Evaluating

#### Questionnaires

**Fig. 5:**
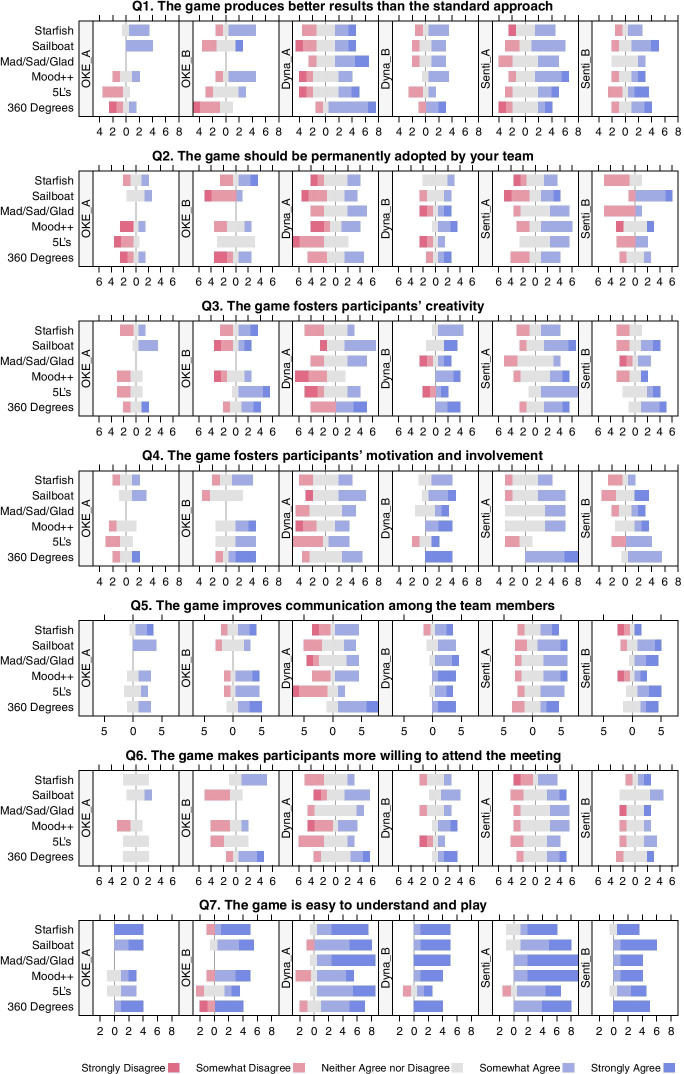
Summary of responses for each question by team. The neutral responses (i.e., “Neither Agree nor Disagree”) are displayed in gray; while negative responses (i.e., “Strongly Disagree” and “Somewhat Disagree”) and those positive (i.e., “Somewhat Agree” and “Strongly Agree”) are shown in shades of red and blue, respectively. The width of a colored bar is proportional to the number of the corresponding responses

The responses for each question are presented with diverging stacked bar charts (Fig. 5) as proposed by Robbins and Heiberger (2011). From the charts, we can see that Dyna_B was the most enthusiastic about the idea of retrospective games, while Dyna_A and OKE_A, despite being the biggest skeptics in general, still found games that improved their retrospectives (e.g. Sailboat for OKE_A). We can also observe that opinions usually vary significantly within each team. Moreover, it is apparent that some teams did not like some games and therefore graded them low in almost all aspects even though individual team members had different perceptions. Specifically, OKE_A disfavored 5Ls and Mood++, OKE_B disfavored Sailboat, while both Dynatrace teams disfavored 5Ls.

Although overall, the retrospective games positively affected all evaluated aspects of the retrospective meetings (Q1, Q3–Q6), the improvements might seem to be rather slight at first sight. Nevertheless, even if a game positively affects the performance of just a few participants, the whole team usually benefits from the ideas put forward by those participants. The aspect that improved the most was “communication” (Q5), while the games that were favored in all aspects by most of the teams were Sailboat and 360 Degrees Appreciation.

Surprisingly, some games received single “strongly disagree” responses even for “communication” (Q5) or “easiness to play” (Q7). Accordingly, we decided to look into the individual questionnaire sheets and analyze them jointly with our hand-written notes taken during the participant observations to find an explanation for the low scores. In OKE_A, the participant who understated the results was a programmer who was probably not aware of the added value of retrospectives due to his low commitment to the project (he was engaged in another project at the same time). As for OKE_B, the tester was an older man who did not speak English. Usually, he had difficulty in comprehending the names of the categories, so he did not like the idea of playing the games and he gave low scores. In both SentiOne teams, there were individuals who did not like some games and rated them low in most aspects. In Dyna_A, two experienced developers evinced a negative attitude toward retrospective games from the very beginning and their attitude was reflected in the scores. They said that code writing is their job rather than debating over and over again. They believed that anything other than coding is just a waste of time. On the other hand, in Dyna_B, one team member, who was very knowledgeable in Scrum, was also very keen on running perfect retrospective meetings with the help of retrospective games. However, if he did not like a game, he graded it low, especially in comparison with his teammates. Accordingly, a “disagree” response does not necessarily mean that the game had a negative impact. It may also mean that the respondent disliked the game or did not find a positive impact regarding the evaluated aspect.

When it comes to Q2, the question turned out to be confusing and some team members interpreted it in a different way than we expected. What we meant by “permanently adopt the game” was to include the game in a toolbox of possible retrospective activities. As for Q6, most of our team members had already been willing to attend retrospectives, so this is probably the reason why “Neither Agree nor Disagree” was the most common answer regardless of the game.

**Fig. 6:**
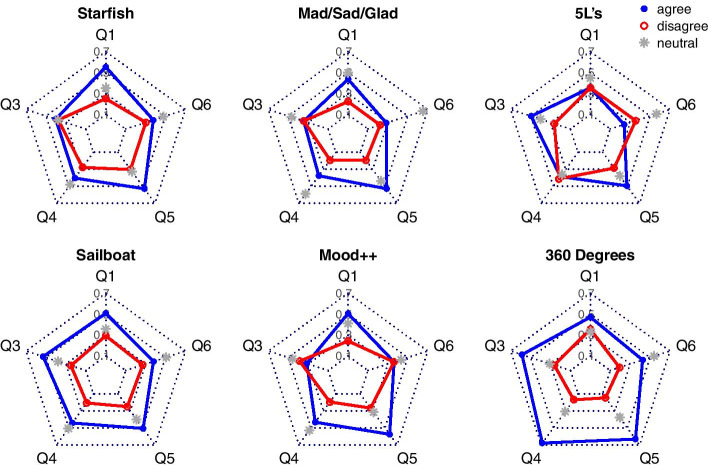
Spider charts of the perception of the adopted games. The answers from all teams are aggregated, giving the larger teams more weight than the smaller ones. Blue filled circles refer to the fractions of agree responses, red circles to the fractions of disagree responses, and gray stars to the fractions of neutral responses

A further examination was made from the perspective of the games by using spider charts (Fig. 6). For each game and statement, we calculated the fraction of participants who “somewhat agree” or “strongly agree” as well as those who “somewhat disagree” or “strongly disagree,” hereafter jointly referred to as “agree” and “disagree,” respectively. Since all games were considered easy to understand and play by almost all respondents (Q7), we did not visualize this category. Likewise, we did not visualize Q2, because it was understood differently by different team members.

Figure 6 shows that 360 Degrees Appreciation performed the best overall. Nevertheless, since this game is not a self-contained retrospective activity, it has (except 5Ls) the worst ratio of positive to negative responses for Q1, which states that the game produces better results than the standard approach. Among the other games, Sailboat, Starfish, and Mad/Sad/Glad still performed quite well. Sailboat has the best ratio of positive to negative responses overall, Starfish has the best ratio for Q1, while Mad/Sad/Glad has the fewest negative answers. Furthermore, 5Ls is dominated by Sailboat, because for each aspect, the latter has a better ratio of positive to negative responses. Finally, all of the games performed well as for “communication” (Q5).

#### Focus groups

To generate more understanding on the outcomes and gain deeper insights into the experiences and opinions of the participants, each internal researcher conducted a focus group (Kontio et al. 2008) with each team to which he/she was assigned. The meetings took place at the office of the participating companies, lasted about 30 min, and all team members were present. Each session started with an introduction from the facilitator about the purpose and process of the session; guidelines on how the participants should discuss; and how the results would be used. Special emphasis was given to assure participants of the anonymity of the discussions (no one will be identified with any comments or findings that are reported) and to make them aware about the importance that their opinions represent the real situation. Six questions were prepared (see Appendix 2) to ensure that the appropriate topics were covered and the discussion stayed focused. Audio recordings were made of all of the focus group sessions for transcription.

An initial analysis of the transcribed material revealed that for every session, the answers to each question were scattered through the whole discussion. The material was then processed using a deductive content analysis (Elo and Kyngäs 2008; Mayring 2014). We started with a set of predefined themes. Each theme corresponded to one of the six questions that guided our focus groups. Furthermore, we defined three categories of codes: *team codes* to refer to the team that generated the statement; *object codes* to point out the object concerned by the statement; and *description codes* that characterize the object. Each category contained a preliminary set of codes, which was extended during the analysis. The final sets of codes were (underlines denote codes that were developed during the analysis):

- team codes: all teams, OKE_A, OKE_B, Dyna_A, Dyna_B, Senti_A, Senti_B
- object codes: game-based retrospective, questionnaire results, all games, Sailboat, Mad/Sad/Glad, Mood++, Starfish, 5Ls, 360 Degrees Appreciation
- description codes: advantage, disadvantage, recommendation, explanation, like, dislike, improvement, declination, surprising result, unsurprising result, discovery, preference.

We analyzed the transcription to identify units of meaning and coded each unit with at least one code from each category. To facilitate coding, we used Microsoft Excel. Each unit was placed in a single row in the first column. We used a separate column for each of the three categories of codes (i.e. team codes, object codes, description codes). Table 8 provides some illustrative examples of coding of the discussion in Senti_B when answering the second question. Next, we developed a central descriptive narrative of the phenomenon under study for each focus group question. Ultimately, the outcome of our content analysis was enriched with examples coming from the participant observation in order to back up the findings. The final result is presented in the subsequent discussion. Note that during the focus groups, when someone expressed an opinion, some other participants either agreed with it, possibly adding something from themselves, or voiced different opinions. In either case, usually there were a few participants who needed encouragement to voice their opinions; so, we asked the question – “What is the opinion of the rest of the team members?” If the great majority of team members agreed on a particular view, it is presented as a team-level statement in the discussion below.

**Table 8:** Coding example

unit of meaning	team code	object code	description code
The approach “different game at each meeting” is not good, because I do not like if something changes and there is a need to learn from scratch. {Another person agreed on this opinion.}	Senti_B	game-based retrospective	dislike, preference
It is worth changing the game after a longer time of usage. {A few people agreed on this opinion.}	Senti_B	game-based retrospective	recommendation
One game was unusual. It was about praising each other. {The facilitator recalled that it was 360 Degrees Appreciation.} This game is cool, because it gives a different view, and it is easy to understand.	Senti_B	360 Degrees Appreciation	like, explanation
However, if we ran this game all the time, that wouldn’t be good. This game is worth conducting once in a while.	Senti_B	360 Degrees Appreciation	recommendation
The variety of games baffled us.	Senti_B	game-based retrospective	dislike
It’s okay to change games from time to time, because different teammates have different preferences. If we stick to one game, some teammates may feel excluded.	Senti_B	game-based retrospective	recommendation
It’s worth conducting the same game for a few sprints to fully understand it and to memorize its rules and then switching to another game. {All teammates agreed on this opinion.}	Senti_B	game-based retrospective	recommendation

1. *How do you assess the new way of running retrospective meetings,* i.e.*, by playing a retrospective game?*

All of the teams except for OKE_B enjoyed game-based retrospectives and considered the new approach valuable even though there were single opposing voices. OKE_A agreed that the games allowed them to improve their work efficiency as well as the Scrum process. Among other things, they started to run the Daily Scrum; they decreased the pull request evaluation latency by granting the approval permission to another programmer; the availability of the Product Owner for the development team was increased; the Product Owner became aware of many problems that the development team faced; the need to hire a graphic designer was identified (this need was met shortly after the research process was completed); MVC controllers were refactored to REST API with Angular. Finally, playing the games also created a sense of community in the team.

OKE_B was the only team in which the new approach did not bring much value since most of the game sessions ended with a similar conclusion, i.e., the main problems were due to a communication gap between the Product Owner and the Development Team. Taking into account that the team worked under time pressure, playing games was considered too time-consuming. Furthermore, the Scrum Master viewed most of the games as too childish. Nevertheless, the team agreed that retrospective games should be played at times, for instance every third sprint, because the games still helped them to generate novel insights, e.g. how to customize JIRA (shortly before our research started, the team underwent a migration from TFS to JIRA).

As for Dyna_A, two team members would prefer not to use retrospective games in the future. They believed that similar conclusions could be made in the course of standard retrospectives, so there was no point in learning retrospective games. However, the rest of the team members were in favor of the new approach. The team also suggested exploring new retrospective games, such as *Three little pigs* and *SWOT.*^1^ They said that playing the same game over and over brings similar comments, but when a new game with different categories is introduced, it makes the participants think more deeply and results in novel outcomes. As for Dyna_B, all team members were uniformly positive about the overall experience.

In both SentiOne teams, only one team member did not like game-based retrospectives. Senti_A said that the new way of running retrospective meetings gets participants engaged and stimulates the imagination, which in turn generates more ideas to discuss. Thanks to retrospective games, they began to specify backlog items more accurately and made developers be more involved in testing. Senti_B agreed that each game improved the meetings as well as the teamwork regardless of whether it was liked or disliked by someone, because it allowed them to elicit insights in a systematic way. They also concluded that they should conduct retrospective meetings, but not necessarily after each sprint.

2. *Should we use the games alternately from one meeting to another, or should we choose any particular game to play all the time?*

Both OKE teams, Dyna_A and Senti_A said that different games should be played interchangeably, because playing the same game over and over would be boring, while playing a variety of games increases the chances of identifying a variety of issues. In contrast, Dyna_B and Senti_B concluded that they would prefer to play the same game for several sprints to fully comprehend it. Only after this learning period, they would like to switch to another game. Senti_B also noticed that using the games alternately is desirable, because team members have different preferences, so it would be hard to choose one game that satisfies everyone.

3. *What are your comments on the results (at that time, we presented a summary of the responses using diverging stacked bar charts)?*

OKE_A said that a game board in the form of a table is not attractive, so Mood++ and 5Ls received low scores. OKE_B claimed that in their project, the goal, threats and problems usually remain the same. Accordingly, playing Sailboat more than once would generate very similar results, thus they graded the game low. Both OKE teams agreed that 360 Degrees Appreciation produces worse results than the standard approach, because it forecloses a full retrospective. Consequently, they graded the game negatively regarding Q1 and Q2, even though they appreciated the positive influence of the game on the other aspects. In contrast, Dyna_B gave 360 Degrees Appreciation high scores because it met their expectations – they always left the meeting in a good mood.

Team members of Dyna_A and Senti_A were surprised that Sailboat was assessed moderately, while, in fact, they liked the game as well as drawing its game board. For Dyna_A, action items generated during the game also helped the team to elaborate a systematic approach to analyzing and prioritizing bugs reported by customers. When it comes to Dyna_B, they liked Sailboat so much that they were surprised that someone graded it negatively with regard to Q1 and Q2.

Some Dyna_A members were also surprised that 5Ls received low scores even for communication (Q5), since the game not only helped them to identify problems in communication within the development team as well as between the team and the Product Owner, but also to solve these problems. However, other Dyna_A members as well as Dyna_B were not surprised and stated that this was due to the fact that 5Ls has weird and confusing categories.

Senti_B said that the scores would have been better if they had played the same game continuously for several sprints and only then moved on to the next one.

Finally, some Dyna_A members admitted that they had a negative attitude toward the research, because they knew that they were going to be moved to other teams. Accordingly, they had no interest in improving the teamwork, so they did not put much effort into the assessment.

4. *Are any of the games particularly useful in some situations/circumstances?*

OKE_A said that 360 Degrees Appreciation is a great way to acknowledge programmers’ efforts and boost the motivation, while OKE_B and Dyna_B suggested that the game seems to be suitable during the forming stage of team building, when members are discreet with their behavior. Besides this, Dyna_B switched to a standard approach after the game finished in order to continue the retrospective and to develop action items. In turn, most of the Dyna_A members had been reluctant to play this game. However, after the first session, they admitted that the game resulted in a better atmosphere. Moreover, they said that the game is particularly useful when there are misunderstandings or arguments between team members. During one of their implementations of that game, they found out that some tensions between them were due to different approaches to developing a particular feature. Thanks to the game, they also agreed on a solution that combined both approaches. Nevertheless, all teams agreed that 360 Degrees Appreciation should be used less often than other games, for instance, every two months.

Both Dynatrace teams also suggested that Mad/Sad/Glad and Sailboat are suitable to start the adventure with retrospective games, since they are easy to learn and play. In Dyna_A, playing Mad/Sad/Glad revealed that communication between them and another team that was supposed to help with some part of the project was inadequate because of the other team. As an action item, the team leader started to talk to that team, which resulted in better communication and a faster development process. Moreover, Dyna_B recommended using Starfish when the team wants to discuss a specific issue in a systematic way. This game helped them to realize that they had wasted too much time on up-front analysis and planning instead of implementing a feature and then discussing it with the users. Thanks to Starfish, they shifted into more agile ways of developing software. Subsequently, they figured out that some features that they had considered as worthless, in fact, had only been used in a wrong way.

When it comes to Senti_A, they stated that it is difficult to choose one game over another because all of them except 360 Degrees Appreciation are of the same type with just slightly different formats.

5. *What are the advantages and disadvantages of each game and why did some games perform better than the others?*

Answers to this question were given throughout the whole meeting. Recurring opinions are summarized in Table 9. There were also some individual opinions, e.g. “Sailboat prevents team members from expressing their personal feelings on what they liked or disliked about the Sprint,” but we discarded them. Interestingly, the Dynatrace teams suggested extensions to the games they used during the research. They proposed converting 360 Degrees Appreciation into 360 Degrees Feedback by allowing for negative comments. In addition, Dyna_B proposed extending Sailboat and Starfish with an *Appreciation* category.

**Table 9:** Advantages and disadvantages of six retrospective games

Game	Advantages	Disadvantages
Sailboat	- clear, distinctive categories - uses metaphors to stimulate creativity - helps team members to align goals	- may become boring if used too often, because the vision and risks rarely change through the project
Mad/Sad/Glad	- lets bad emotions and toxic feelings out - simple rules - not time-consuming	- *Mad* category is too similar to *Sad* category - restricts the discussion
Mood++	- makes team members kinder toward each other - allows the team discuss any ideas	- time-consuming
Starfish	- names of the categories explicitly call to actions	- sometimes it is not obvious to which category an idea should belong - time-consuming
5Ls		- names of the categories are confusing for non-English natives - fuzzy boundaries between the categories - time-consuming
360 Degrees Appreciation	- strengthens team relationships and trust - fosters self-esteem - alleviates conflicts	- a team building activity rather than a Sprint retrospective - precludes constructive criticism

6. *Have retrospective games helped your team mitigate some of your retrospective problems?*

At this point, we asked each team to look back at the retrospective problems they had encountered before our project started (Table 7) and consider whether retrospective games helped them to mitigate the problems. All of the teams indicated that the introduction of retrospective games only partially solved the “little added value” problem. Actually, we expected such answers. As a side effect of our project, we found out that the main causes of reducing the frequency of retrospective meetings were not the retrospective itself, but organizational dysfunctions. Issues such as difficulties in communication between developers and the Product Owner or between different teams, forcing teams to overload the Sprint Backlog, and assigning people to multiple Scrum teams were raised during retrospectives before our project started, but nothing was done since the solution would either violate the corporate governance or required corrective actions at the organizational level. This impossibility caused frustration, which in turn caused teams to skip retrospective meetings. We also did not try to deal with these issues, since it was out of the scope of our research and the agreement with the host organizations on carrying out the project did not authorize such intervention.

On the other hand, we expected our intervention to solve other retrospective problems faced by the teams. However, only one problem, namely “unequal participation,” was fully solved in all of the teams. As for the other problems, they were mitigated to some degree. Dyna_B and Senti_A benefited the most from the new approach to the Sprint Retrospective since 3 out of 5 identified problems were fully solved. Note that although Senti_A originally believed that their participation in the discussion had been even, they acknowledged that retrospective games improved the situation. Besides this, Dyna_A claimed that the introduction of games did not reduce the number of complaints, but this was not a problem because their complaints usually revealed actual problems, while our approach allowed them to see the importance of the problems (e.g. when all team members wrote almost the same comment). Consequently, it was easier to decide which problems should be tackled first.

### Specifying learning

In this section, we derive eight lessons learned based on the findings from the interviews (Section 5.1), questionnaires (Section 5.4.1), focus groups (Section 5.4.2) as well as our impressions from the participant observations. The sources of evidence for each lesson learned can be traced in Table 10. We believe that our lessons will be useful for practitioners interested in improving their retrospectives as well as for other researchers interested in investigating other collaborative games. In short, they suggest that game-based retrospectives are usable and useful even though not everyone likes them.

**Table 10:**
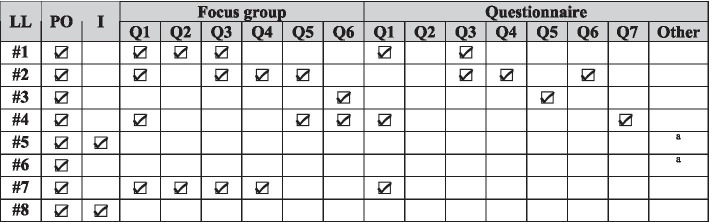
Sources of evidence that inform our lessons learned

*LL* Lesson Learned, *PO* Participant Observation, *I* Interview, ^*a*^ Analysis of individual questionnaire sheet

#### Lesson 1: Retrospective games provide meeting diversity, which encourages new perspectives and helps teams bring up new ideas

By changing the way retrospectives are run, not only retrospective games break the habitual routine, but they also encourage teams to look back at the Sprint from varying viewpoints. In our experience, the former prevents anyone to be half-asleep (all participating teams occasionally experienced such situations during traditional retrospectives), while the latter allows teams to generate new insights, uncover new opportunities, and identify novel solutions. The overall result is that more ideas are generated. Nevertheless, according to the Cognitive Network Model (Santanen et al. 2000), people always like to use familiar solutions to resolve the problems they encounter. Our experience confirms that introducing a new game at every meeting may be overwhelming for some teams, hence we advise Scrum Masters to ask the team whether they are ready to learn a new game or would prefer to continue practicing the previous one(s).

#### Lesson 2: Retrospective games drive participants into a collaborative mindset, but do not lead to breakthrough findings

In our view, although retrospective games have not proved to be enjoyable enough to trigger a flow state, they still keep participants more engaged and focused as they require participants to perform a specific task at each stage of the meeting. Moreover, according to our observations retrospective games elicit fun (participants smiled more often) and positive emotions (there was no mutual hostility even if difficult issues were discussed), which improves work satisfaction, breaks down barriers, and drives participants into a collaborative mindset. Consequently, more ideas are brought out. Furthermore, the closer and more intensive interaction between team members is a good way to socialize, and improves the team spirit (it was much easier to find volunteers to pursue action items). Unfortunately, even though playfulness and interaction are central in stimulating creativity, our findings suggest that the role of retrospective games in discovering speculative breakthrough opportunities is exaggerated.

#### Lesson 3: Retrospective games encourage equal participation

As retrospective games require each participant to write down his/her ideas on post-it notes, it is our experience that they not only challenge less vocal people who do not feel comfortable raising issues, but also some participants with passive attitude to take a more active role. As for the former, they appreciated that finally they had an opportunity to voice out. In turn, when it comes to participants with passive attitude, the majority were activated by games, but two individuals with a negative attitude toward retrospective meetings still remained withdrawn. Besides, by encouraging equal participation, retrospective games seem to contribute to the divergence of views, which in turn creates the potential for constructive controversy. Overall, as a result of game-based retrospectives, more ideas are contributed and more opinions are voiced out.

#### Lesson 4: Retrospective games organize both the meeting as well as the discussion

Retrospective games provide structure for retrospective meetings and focus the discussion. As a consequence, we observed a paradigm shift from complaint-orientation to constructive solution-oriented discussions. Before implementing retrospective games in Dynatrace as well as SentiOne teams, barren discussions and complaints sometimes went on so long that there was not enough time to discuss all essential problems, thoroughly identify the sources of the problems or propose action items. Thereby, there was often an impression of unfinished discussion. Thanks to retrospective games, it was easier to identify essential problems (usually those that were raised by a number of post-it notes) and discuss them in the context of their potential resolutions, which enhanced the meeting productivity. On the other hand, in both OKE teams, game-based retrospectives usually lasted longer than their traditional counterparts as the discussions were much more comprehensive. Nevertheless, this may be considered as a disadvantage by some traditional managers especially if they control the team with the help of metrics.

According to our observations, retrospective games also make it easier to facilitate the meeting and reduce the preparation effort. Indeed, to some degree, game-based retrospectives are self-facilitated. For instance, during the second round of game-based retrospectives, when all the rules and steps were known, different developers voluntarily took over the role of a moderator and facilitated the meeting. This approach was especially welcomed by Dyna_A, whose some team members had complained that their Scrum Master had overcontrolled the retrospective meetings.

#### Lesson 5: Not all agile team members have an agile mindset

Based on our experience, we think that developers who perceive retrospective meetings as a waste of time probably will not change their minds after implementing retrospective games, especially if their companies focus too intently on short-term efficiency gains (indeed, in all participating teams, retrospective meetings were sometimes canceled or postponed because of a more important meeting or the time pressure to complete the work). Furthermore, retrospective games may be perceived as childish and be embarrassing for reserved people. Indeed, some participants who considered themselves as “serious developers” insisted that they “could have contributed more to the project by coding instead of playing retrospective games”. As such, we believe that it is better not to force them to take part in retrospectives, because they may bring negative energy and even sabotage the meeting. However, agile coaches may perceive our view to be too simplistic and even dangerous as conducting a retrospective without the whole team present goes against the Scrum Guide. Accordingly, this issue requires further investigation. We also found that there are usually some individuals who do not contribute to the meeting, but take a free ride on those who do contribute. Probably, the best solution for both issues would be to find the right people – those who can leverage agile practices in the way that they are intended – at the stage of team building. Our discussion may be supplemented by a reference to Ellis et al. (2014) who stated that the effectiveness of systematic reflection depends on person-based factors and is higher for learning-oriented people who are conscientious and emotionally stable, can accurately evaluate their performance, and enjoy effortful cognitive activity.

#### Lesson 6: If participants are not afraid to voice their opinions, data obtained through a focus group is more reliable than through a questionnaire

In our judgment, the feedback on our intervention obtained during the focus groups is more positive than that obtained through the questionnaires. The reason for this difference is probably that opinions put forward during a focus group must be supported by a reasonable explanation and hence they must be genuine. On the contrary, if no justification is required in a questionnaire, it is likely that some participants may try to sabotage the results or they simply do not reflect deeply on the results.

#### Lesson 7: Scrum masters should have a toolbox of possible retrospective games and help their teams empirically determine which games are effective for them

The variability in the responses of individuals as well as the teams to the games is quite high. This means that what works for some may not work for others and that the preference of the game probably depends on the personality of the person. Nonetheless, some general findings on the advantages and disadvantages of each investigated game are presented in Table 9, while below we present further guidelines for Scrum Masters. In our view, Sailboat is a good choice for teams who are starting their adventure with game-based retrospectives. It is simple, but it keeps all team members pulling in the same direction and helps them think outside of the box, which results in fresh new ideas. Besides, drawing a sailboat together promotes a sense of community. However, after a few sessions, we advise teams to switch to Mad/Sad/Glad or Starfish, because otherwise Sailboat may become boring. Mad/Sad/Glad has the most straightforward rules and the quickest play time. In contrast to Sailboat, it allows the team to discuss the past Sprint from the feeling perspective and release negative emotions. Nevertheless, according to the participants’ experience, the game does not cover all of the topics that should be discussed during a retrospective, while running the game does not directly generate constructive suggestions for improvements. Mood++ addresses the latter and provides an opportunity for team members to compliment each other. In contrast to Mad/Sad/Glad, Starfish covers all aspects of the traditional agile retrospective and allows for a straightforward application of the findings, as stated by some participants. Another game that also covers all aspects of the traditional agile retrospective is 5Ls. However, according to our findings, its category names may cause confusion and anger among participants, so we do not recommend it to teams who have just started running game-based retrospectives. Finally, a completely different type of game is 360 Degrees Appreciation. It helps the team to loosen up a bit and relax after a stressful sprint. It is our experience that the generated feedback is usually enthusiastically welcomed and boosts the team spirit. Nevertheless, the game is not a standalone retrospective technique.

#### Lesson 8: Organizational culture and managers’ mindsets are still significant barriers to the successful adoption of agile practices

Our observations agree with findings of other researchers (Nerur et al. 2005; Fruhling and Tarrell 2008; Cao et al. 2009; Hanslo and Mnkandla 2018; Gupta et al. 2019; VersionOne, 2020; Spiegler et al. 2021) that organizational culture is a significant barrier to the successful adoption of agile practices especially for bureaucratic organizations steeped in plan-driven methods. Due to the tayloristic past, project managers tend to stick with traditional viewpoints on hierarchy in terms of reporting, resource allocation, decision-making, etc. and are reluctant to grant leadership roles to the teams (Moe et al. 2012; Spiegler et al. 2021). However, agile methods require a shift from command-and-control project management to shared decision-making and self-management teams, where project managers are facilitators who coordinate collaborative efforts and create a supportive organizational context (Nerur et al. 2005; Dybå et al. 2014b; Theobald et al. 2020). Therefore, in order to enjoy the full benefits of Scrum, agile values and principles must be adopted not only at the team level but also at the organizational level.

Moreover, we found that continuous process improvement is hard to achieve even though inspect-and-adapt cycles and reflection are central concepts in agile development. In 5 out of 6 teams participating in the project, the implementation of Scrum deviated from the framework. In all cases, the deviations covered up organizational dysfunctions which, if addressed and removed, would allow the team to be more effective and efficient. What is more, in our opinion such dysfunctions can be discovered in the course of traditional retrospectives. In turn, the main reason for reducing the frequency of retrospective meetings was not the way in which the meetings were run, but unproductive discussions on recurring problems that were beyond the control of the team (e.g. unavailability of the Product Owner for requirements clarification, lack of communication between Scrum teams, or distribution of team members across multiple rooms). Indeed the phenomenon of repeating discussions was also experienced by teams investigated by Lehtinen et al. (2017). This again indicates that making the transition to the agile mindset (Schön et al. 2017; Miler and Gaida 2020; Özkan et al. 2020) and understanding agile values is still challenging for some companies. Taking into account that we were also not authorized to fix organizational dysfunctions (the scope of our agreement was limited to improving retrospectives), we decided not to start a new AR cycle, because we believed that this would not bring much added value.

## Threats to validity

Although the concept of research validity has been discussed widely in the context of controlled experiments, surveys, and case studies, as for AR, the literature is very scarce. Admittedly, Baskerville and Wood-Harper (1998) identified seven validity criteria for AR in Information Systems, but their set of criteria is not in line with any of the well-known validity systems and thus it is probably difficult to use. No wonder action research studies do not typically discuss possible threats.

Recently, Staron (2020) proposed a validity system for AR in the context of software engineering, which is based on the taxonomy by Cook and Campbell (1979). Accordingly, his taxonomy distinguishes between four types of validity, i.e. construct, internal, external, and statistical conclusion validity. However, the campbellian validity system was designed from the viewpoint of quantitative research; thus, in order to be adopted by action researchers, it must be refined to include one more aspect of the validity, namely reliability, which has been commonly used in qualitative research such as case studies (Yin 2018). Thereby, in the context of AR, we propose to evaluate the validity from five perspectives that can be summarized as follows:

- **construct validity** reflects to what extent the operational measures truly represent the underlying realities of the intervention;
- **internal validity** describes the extent to which the observed effect (intervention outputs) is caused only by the intervention;
- **external validity** concerns to what extent the results of the study can be applied to other settings, and to what extent the results are of interest to other people outside of the participating organizations;
- **reliability** considers the extent to which the data and the analysis are dependent on the specific researchers;
- **statistical conclusion** validity refers to the appropriate use of statistics to infer about the correlation between treatment and outcome.

In the following subsections, we discuss the validity of our research from the first four perspectives. The fifth perspective, i.e., the statistical conclusion validity, is not relevant to our research as we did not employ inferential statistics.

### Construct validity

The main limitation of our study is that the impact of retrospective games was evaluated based on the perceptions, feelings, experience, and memory of the participants rather than more objective measurements (e.g., the number of evidence-based valuable ideas generated). However, objective measurements would be useless in our study. Each sprint is unique; thus even conducting retrospectives with the same method must generate different outputs for each sprint.

Relying on human input creates several social threats to construct validity. As the participants were pre-informed about the aim of the intervention as well as the kind of improvements we expected, they could have adjusted their behavior accordingly or answered what the researchers wanted to find (Staron 2020). Moreover, some people tend to provide positive feedback regardless of their real opinion (Robson 2002). To mitigate the social threats, all participants were informed up-front that only honest responses were useful for us because we wanted to filter out those games that do not improve retrospective meetings. Besides, during the focus groups, the participants were encouraged to criticize the games and to point out the difficulties and limitations faced by them when running the game-based retrospectives.

Another limitation related to the trustworthiness of the participants is that they could have intentionally tried to influence the results to sabotage the study. For instance, those who hated retrospective meetings might have been interested in findings that retrospective meetings are not productive regardless of being run in the traditional or game-based approach. In addition, some individuals just did not put much effort into giving thoughtful feedback.

Apart from the gaming activity, we also introduced other improvements to retrospective meetings (e.g., a hybrid brainwriting/brainstorming approach). Accordingly, the “real” intervention that the teams received was actually a combination of introducing both (1) retrospective games; and (2) the generic model of orchestrating the flow of information and ideas in the course of the game. Furthermore, in both OKE teams, our main intervention was taken in parallel with other improvement activities. In OKE_A, the joint Sprint Review/Retrospective meeting was split into two separate meetings, while in OKE_B, the Sprint Retrospective began to occur regularly. Nevertheless, OKE_A turned out to be the greatest skeptics, while the results from OKE_B did not significantly differ from the others.

Another potential threat to construct validity is researcher expectancy bias, meaning that we could have interpreted evidence collected according to our expectations and beliefs.

Lastly, a misunderstanding of the questionnaire statements could have influenced the responses. Actually, this was the case for statement Q2 and thus we did not interpret the corresponding responses, which is a limitation of our study.

To mitigate all of the aforementioned threats, we also utilized investigator, data source, and method triangulation (Denzin 1970; Cook and Campbell 1979; Jick 1979; Davison et al. 2004). Three different investigators collected the data from three different companies using interviews, questionnaires, focus groups, and participant observations.

### Internal validity

Whenever conducting research in a real-life industry setting, not all variables can be controlled. The main threat to internal validity is that factors other than our intervention could have influenced the results. Firstly, different runs of a game naturally produce richer or poorer outputs depending on what has happened in the sprint as well as the commitment and mood of the team members in the reflection process (Poth and Riel 2020 have observed that participants’ moods and engagements heavily influence the quality of the brainstorming session). Secondly, there were a few developers who hated the meetings and so gave low scores to all games. If such a developer was absent, the game automatically avoided one “disagree” response. In order to reduce both of these threats, we recommended implementing each game twice.

Thirdly, the order in which the games were introduced could also have biased the results, e.g. in favor of games that were introduced later when the generic flow orchestration model was known or in favor of the first presented game due to the novelty effect. To reduce this threat, we did not suggest the order in which the games were introduced but left this decision to the meeting facilitators. In this way, the order was different for each team.

Fourthly, the facilitators of the retrospectives varied among the teams. There is a threat that the facilitator involvement influenced team members in generating more ideas, which in turn affected the outcome of the retrospective meetings. However, variation in the facilitators is unavoidable in a study conducted in three companies.

Fifthly, the diagnosing phase might have biased the participants because we asked about the problems with their retrospectives and therefore we indicated that the practice needed to be improved.

Sixthly, our participants were subject to Hawthorne Effect, i.e., they might have improved their behavior simply because they were being observed.

Finally, as action research takes a longer period of time, people join or quit teams, which biases the comparison before and after the intervention. Indeed, we lost single participants during the project.

### External validity

The problem of generalizability from single cases is a common and recurrent problem in industry (Sjøberg et al. 2007b; Ghaisas et al. 2013). Wieringa and Daneva (2015) argue that the variability of the real world implies that we will never have universal theories in software engineering. At the same time, they emphasize the utility of middle-range theories that balance generality with practicality. Practitioners who want to apply a middle-range theory to their particular case should evaluate whether the theory is true for their case, or perhaps needs to be adapted (Wieringa and Daneva 2015).

Generalization from an AR project depends on contextual similarity and is usually referred to as transferability (Meyer 2000). A prerequisite for transferability is a rich and relevant context description. Thereby, much attention in our paper is devoted to this. Not only did we report all steps of our AR project as well as the procedure to conduct a game-based retrospective, but also the interview protocol, focus group questions, and questionnaire items which we used in the different phases of our research. We believe that the provided details will allow other researchers or practitioners to easily replicate our study in another organization.

The key question is to which contexts our findings are applicable. To conduct our research, we chose a context which was suited to the adoption of retrospective games, i.e., all participating teams had experienced common problems with retrospectives. Therefore, our findings are not representative for teams that do not encounter obstacles to effective retrospective meetings. Besides, 5 out of 6 participating teams suffered organizational dysfunctions. Furthermore, our intervention was implemented in on-site teams that conducted face-to-face meetings. As observed by Griffin (2021) conducting an effective remote meeting is more challenging than a face-to-face one. Thereby, our findings are also not representative for distributed teams that run online remote retrospectives. Nevertheless, the concept of analytic generalization allows us to claim that our findings are generalizable to teams using any agile method, even though we studied only Scrum teams.

We are aware that a replication of our study for different teams will likely lead to different game profiles simply because the new participants will have different preferences. However, the approach itself is systematic and generic; a team experiencing problems with retrospectives may follow our procedure to find retrospective games that suit them best and improve their retrospective practice. Besides, we believe that the lessons learned that we draw are directly applicable to other agile teams with similar contexts to ours.

### Reliability

An important question in qualitative research is whether the findings are consistent with the data collected. In order to improve reliability, we developed and reported both the interview protocol as well as the focus group protocol. All focus group sessions were audio recorded and then transcribed. The transcripts from each host company were first independently coded and analyzed by the internal and the external researcher (investigator triangulation), and then the results were jointly discussed while the differences in coding were resolved. We also provided information on how to code the collected data. The final narration was proposed by the external researcher and reviewed by and agreed with all internal researchers.

Nevertheless, some of our findings are based on data collected in an ad hoc, unsystematic manner. Each internal researcher as an employee of one of the host companies, often received spontaneous feedback on the intervention during their daily work on different occasions (e.g. chatting during lunch time and breaks). Because such chats were unplanned and initiated by the teammates, they did not follow any protocol. This kind of feedback was then documented superficially as the internal researchers were usually busy with their professional duties and responsibilities in the workplace. Although this approach to data collection is something inevitable during participatory observation, which is an inherent part of AR (Baskerville and Wood-Harper 1998), it compromises the reliability since the process of data collection and analysis cannot be repeated in exactly the same manner.

## Implications for research and practice

This study extends the body of knowledge on the Sprint Retrospective. Prior literature has mainly focused on: (1) the process and steps of a retrospective (Derby and Larsen 2006; Rubin 2012; Kua 2013; Andriyani et al. 2017; Schwaber and Sutherland 2020; Loeffler 2017; Mas et al. 2018); (2) specifying the techniques of conducting a retrospective meeting (Gonçalves and Linders 2014; Roden and Williams 2015; Krivitsky 2015; Lehtinen et al. 2015; Caroli and Caetano 2016); and (3) recommendations on how to make retrospectives effective (Babb et al. 2014; Lehtinen et al. 2017; Gaikwad et al. 2019; Mesquida et al. 2019; Marshburn 2018; Matthies et al. 2019). However, there is little research evidence on the effects of collaborative games on retrospective meetings. In this paper, we start to fill in this gap. Our research contributes to the awareness of the challenges in conducting successful retrospectives. It also provides practical guidance of how agile teams may mitigate common retrospective problems by introducing game-based retrospectives. Further, we present the advantages and disadvantages of six games and created their profiles to show the potential of each game to stimulate participants’ communication (which was found to be strongly correlated with the effectiveness of agile teams (Ramírez-Mora et al. 2020)), motivation-and-involvement, and creativity. Finally, we provide eight lessons learned that could be of interest for both researchers and practitioners. The knowledge may be used not only by Scrum Masters, but by everyone whose goal is to improve software development processes. We hope that our experience will motivate others to adopt retrospective games in their teams and share the outcomes. With time, the accumulation of new feedback should provide opportunities for identifying additional lessons learned and update the profiles.

Our study also opens up new directions for research. Researchers can use our intervention design to investigate the applicability of collaborative games to other Scrum meetings. For instance, there are several collaborative games for user story estimation, which, to the best of our knowledge, have not been previously evaluated. For those who are interested in continuing our research, we recommend implementing retrospective games in all subsequent meetings. This will make it easier for participants to compare the effects of the adopted games and will increase the chance of evaluating all of the games in the same team composition. We would also like to highlight that the fact that one member of each participating organization became the action researcher, contributing to the success of our AR. It was not only much easier for the researcher to understand the background in which the AR was conducted, but also to get acceptance from the AR participants.

We also make a contribution to the software engineering (SE) body of knowledge regarding the use of AR. Although empirical methods have received much attention in SE research and the community has matured in its use of controlled experiments and case studies, action research seems almost absent in SE research venues. According to Sjøberg et al. (2007a), one of the reasons for this situation is that “the understanding of what Action Research means in the context of SE is little understood.” In 2009, Santos and Travassos (2009) conducted a systematic literature review to identify action research studies in the SE context in the period of 1993 to June 2009. They identified only eight studies that could be classified as genuine action research. Over the last decade, the situation has not much improved, and more recently Garousi et al. (2020) have explicitly called for more AR studies in SE, while Staron (2020) has presented the theory and practice of AR in the SE domain.

However, the “industry-as-laboratory” research concept, which in fact is addressed by Action Research, has been advocated by the SE community for about 30 years. On the 25th-year anniversary of SE, Colin Potts (1993) asked “why most of the research done so far is failing to influence industrial practice” and argued that “an artificial barrier between industry’s problems and the problems that many researchers choose to address” is the main cause for the limited impact of SE research. As a remedy, he called for a paradigm shift from “research-then-transfer” to “industry-as-laboratory” where “researchers identify problems through close involvement with industrial projects, and create and evaluate solutions in an almost indivisible research activity.” Since then, the need for tighter links between academia and industry has been echoed many times by the community. In 1994, Robert Glass (1994) envisioned that in the year 2020 “a researcher is working alongside a practitioner, being open to adjusting and improving ideas in order to make them useful in practice.” 18 years later, Briand (2012) argued that SE research must be carried out in practical settings with collaboration with actual software development organizations. To steer the community toward a more successful future, in his succeeding paper, Briand et al. (2017) appealed for context-driven research, which means research “driven by concrete needs in specific domains and development projects.” They also called for developing guidelines on how to carry out and review context-driven research. Sadly, in this day and age, significant challenges for a successful industry-academia collaboration are still observed (Marijan and Gotlieb 2021).

Being submitted in the year targeted by Glass’ vision, not only does the current paper meet the expectation of “software practice and research work together” (Glass 1994), but also answers Briand et al.’s request (Briand et al. 2017) and provides implicit guidelines on how to evaluate new technologies in context-driven research by demonstrating the usage of AR in SE research and reporting on our collaboration with the industry.

Lastly, we also provided two contributions to AR methodology. We proposed a multiple Action Research design, which allows for better generalization of the findings. Having a broader range of empirical approaches in their arsenal, software engineering researchers are able to pick the most suitable one depending on the research problem at hand. We also refined the validity system for AR introduced by Staron (2020). Given the qualitative nature of AR (Baskerville and Wood-Harper 1998), along with four validities already adopted from the campbellian system (Cook and Campbell 1979) by Staron, our revised system also includes reliability, which we borrowed from the validity system for case study research (Yin 2018).

## Evaluation of our action research project

In this section, we report on how we adhere to the principles of Canonical Action Research by Davison et al. (2004). Not only did the principles help us to assure both rigor and relevance, but they also may be helpful for reviewers who assess the execution and presentation of this research.

### The principle of the researcher–client agreement

During pre-project discussions with the host organizations, we explained the idea of game-based retrospectives and the expected benefits of its implementation. We also presented the AR method and its potential to support parallel academic and practical objectives. The host organizations agreed that the game-based approach and the research method suit the organizational situation and we had a shared interest in running the project. Subsequently, we developed a framework that was mutually acceptable to the organizations and the researchers. The researchers’ responsibility was to guide the overall process. We also obtained permission from management to publish the research results. The host organizations committed themselves to adopting game-based retrospectives within their teams and engaging in a process of critical reflection on the approach under study as well as the research results. The framework then specified the research focus, data collection techniques, and evaluation measures. In the spirit of Agile, we did not sign any contract, but we had a solid word-of-mouth agreement on carrying out the project.

### The principle of the cyclical process model

Our project followed the Canonical Action Research process model defined by Susman and Evered (1978). We started our project with a diagnosis of the situation in the participating teams. The diagnosis directly informed the planning of actions which were subsequently implemented. Then, the intervention was evaluated and analyzed. Finally, we reflected on the project outcomes. Since the specified objectives were achieved, we finished the project after one cycle. Nevertheless, we identified opportunities for future research.

### The principle of theory

We thoroughly reviewed existing theories pertaining to the domain of our project to suggest actions that may be effective and to position the research within the accumulated scholarly knowledge. Consequently, we relied on the theory for diagnosis (the interview protocol was based on common retrospective problems identified by Kua 2013 and Matthies et al. 2019) as well as intervention (flow theory (Csikszentmihalyi 1991), group flow theory (Sawyer 2007), group creative theory (Sawyer 2007), broaden-and-build theory (Fredrickson 1998), social influence theory (Paulus and Dzindolet 1993), and game theory (von Neumann and Morgenstern 1944)). Based on Paulus and Nijstad’s (2003) group creativity model, we proposed a generic model of running a retrospective game. We explicitly paid attention to the relevance of our AR project for the host organizations and agile practitioners in general as well as the research community.

### The principle of change through action

The researchers and the host organizations were motivated to alleviate the retrospective problems that were identified during the diagnosis of the participating teams. As a remedy to the diagnosed problems, the researchers suggested adopting retrospective games. The planned actions were approved by the host organizations. After the intervention was enacted, the degree of problem resolution was discussed, while all research phases and results were properly documented.

### The principle of learning through reflection

Together with the participating teams, we reflected on the outcomes of the project. As a result of our collaborative reflection, we specified eight lessons learned as well as the implications for both further research and practice. Besides this, we refined AR itself by revising its validity system and proposing a multiple AR design, which helps AR researchers to overcome the difficulty in the generalization of AR results.

## Conclusions and future work

In this paper, we report our progress on a long-term research aiming at integrating collaborative games with Scrum. The current findings pertain to the Sprint Retrospective and come from an AR project conducted in three software development companies. Our main interest in this project was to investigate whether the promised benefits of collaborative games are materialized during retrospectives, while the interest of the participating organizations was to revise and enhance their retrospective practices. With this in mind, we formulated four research questions to guide our work: RQ1 – How do game-based retrospectives impact common retrospective problems?; RQ2 – How do retrospective games impact teamwork qualities?; RQ3 – What are the advantages and disadvantages of the adopted games? (answered in Table 9); and RQ4 – What are the lessons learned in adopting retrospective games? (answered in Section 5.5). To answer the questions, we adopted six retrospective games in three organizations and examined how the games could benefit retrospective meetings. The feedback received from six teams indicates that the approach is usable and useful, but the hype around collaborative games is exaggerated.

When it comes to RQ1, retrospective games helped the teams mitigate many of the “accidental difficulties” bearing on the Sprint Retrospective, such as a lack of structure, dullness, too many complaints, and unequal participation. They also made the meetings more productive. As a side effect of our study, we found that the main problem with retrospectives was not the way in which the meetings were run, but unproductive discussions on recurring issues that were beyond the control of the team, while the management was not supportive of corrective actions at the organizational level. On the one hand, this could have discouraged teams from being fully engaged in the retrospective meetings and thereby hindered them from taking full advantage of retrospective games. On the other hand, if organizational-level corrective actions had been implemented, the teams might have avoided some of their retrospective problems without introducing retrospective games.

As for RQ2, different participants perceived different games as having a positive impact on their communication, motivation-and-involvement, creativity, and/or willingness to attend the meeting but there was no single game that would satisfy a whole team. The teamwork qualities that improved the most were “communication” and then “motivation-and-involvement.” As for “creativity,” it was substantially improved by Sailboat, 5Ls, and 360 Degrees Appreciation. Nonetheless, a few developers who were unwilling to attend retrospectives did not change their minds after the adoption of retrospective games. Moreover, the teams generally appreciated the variety in retrospective meetings which allowed them to reflect on the past from different perspectives, but the variety was too great for two teams. Therefore, Scrum Masters should have a toolbox of possible retrospective games, help their teams empirically determine which games are effective for them, and switch the game when the team wishes so. In this way, everyone will have a chance to play their favorite game by which they can contribute the most to the process improvement. We also recommend that teams who have not used collaborative games should start with the Sailboat game, which received predominantly positive feedback.

Although all participating teams intended to keep running game-based retrospectives after the project finished, only 4 out of 6 have managed to do so. Both Dyna_A and Senti_A have used different games interchangeably with regularly held retrospectives after every sprint. Dyna_B have also retained retrospective games, which has helped them to identify and solve a variety of problems, and have been holding retrospective meetings more often since participating in the research. After the research project finished, OKE_A tried all of the games again at least once to develop a better understanding of which games suit them the best. They have been playing retrospective games only when all team members are participating in the meeting. Only OKE_B and Senti_B have not permanently adopted retrospective games. When it comes to OKE_B, the prolonged stagnation in the project made the team lose faith that anything could be improved. In turn, Senti_B had no Scrum Master and no one was willing to facilitate game sessions.

We hope that the reported experience will also inspire other practitioners to leverage retrospective games. We encourage the Agile community to reuse our intervention design in their teams and adjust our initial profiles of retrospective games in the form of spider charts (Fig. 6). To make future research easier, we provide what is called a “reproducible package” (Madeyski and Kitchenham 2017; de Oliveira Neto et al. 2019). The package (*https://github.com/przybylek/retros*) includes the collected data as well as R scripts to aggregate and visualize the data.

Recently, online tools for running remote retrospectives have gained in importance due to the COVID-19 pandemic that has reshaped the way software engineers work (Butt et al. 2021b; Griffin 2021; Marek et al. 2021; Neumann et al. 2021). Thereby, we call for future work to investigate retrospective games in the context of virtual retrospectives with a distributed team. Employing online retrospective tools provides some opportunities. Nunamaker et al. (1987) and Davis et al. (2003) demonstrated that electronic idea generation sessions may reduce the negative effects of evaluation apprehension by providing anonymity to team members as well as production blocking. Accordingly, we have just started a new AR project that aims to introduce anonymity in the idea-generation phase of virtual retrospectives. We expect that anonymity will encourage participants to express their true feelings and critical thinking, which in turn will increase the quality and quantity of ideas generated. Additionally, future work may explore other collaborative games and investigate their application not only to the Sprint Retrospective, but also to other Scrum ceremonies.

## Appendix 1 – Questionnaire for the assessment of collaborative games

Below are eight statements with which you may agree or disagree. Please, provide a response for each statement using a scale of 1 to 5, where:

1 – “Strongly Disagree,”

2 – “Somewhat Disagree,”

3 – “Neither Agree nor Disagree,”

4 – “Somewhat Agree,”

5 – “Strongly Agree.”

### Appendix 2 – Focus group protocol

**Purpose:** to construct a common understanding of the results of the intervention.

**Environment:** the same location as where the retrospective meetings took place.

**Moderator:** the researcher who introduced the games during the retrospective meetings.

**Participants:** all members of the team that participated in the research.

**Format:** a semi-structured “moderated discussion” (Staron 2020).

**Discussion questions:**

1. How do you assess the new way of running retrospective meetings, i.e. by playing a retrospective game?
2. Should we use the games alternately from one meeting to another, or should we choose any particular game to play all the time?
3. What are your comments on the results (at that time, we presented a summary of the responses using diverging stacked bar charts)?
4. Are any of the games particularly useful in some situations/circumstances?
5. What are the advantages and disadvantages of each game and why did some games perform better than the others?
6. Have retrospective games helped your team mitigate some of your retrospective problems?

## Footnotes

1 If SWOT is run using the procedure presented in Fig. 3, it can be considered a collaborative game.

## References

[CR1] Abt CC (1970). Serious games.

[CR2] Amabile TM (1983). The social psychology of creativity: a componential conceptualization. In: Journal of Personality and Social Psychology.

[CR3] Andriyani Y, Hoda R, Amor R (2017) Reflection in Agile Retrospectives. In: In: 18th International Conference, XP, Cologne, Germany. 10.1007/978-3-319-57633-6_1

[CR4] Akarsu Z, Metin ÖO, Gungor D, Yilmaz M (2018) Towards a Role Playing Game for Exploring the Roles in Scrum to Improve Collaboration Problems. In: 18th European Systems Software and Service Process Improvement and Innovation (EuroSPI), Bilbao, Spain. 10.1007/978-3-319-97925-0_21

[CR5] Asch SE (1956) Studies of independence and conformity: a minority of one against a unanimous majority. In: Psychological Monographs, 70 (9, whole no. 416)

[CR6] Avison D, Baskerville R, Myers M (2001). Controlling action research projects. In: Information Technology and People.

[CR7] Avison D, Baskerville R, Myers MD (2007) The structure of power in action research projects. In: Kock N. (eds) Information Systems Action Research. Integrated Series in Information Systems, vol 13. Springer, Boston, MA

[CR8] Avison DE, Davisonb RM, Malaurenta J (2017) Information systems action research: debunking myths and overcoming barriers. In: Information & Management

[CR9] Babb J, Hoda R, Norbjerg J (2014) Embedding Reflection and Learning into Agile Software Development. In: IEEE Software, Vol. 31(4)

[CR10] Baburoglu ON, Ravn I (1992). Normative Action Research In: Organization Studies.

[CR11] Baldauf C (2018) Retromat - Run great agile retrospectives! Leanpub

[CR12] Baruah J, Paulus PB (2008). Effects of training on idea generation. In: Small Group Research.

[CR13] Braun V, Clarke V (2006). Using thematic analysis in psychology. In: Qualitative research in psychology.

[CR14] Baskerville R (1999) Investigation Information Systems with Action Research. In: Communications of the Association for Information Systems, Vol. 2(19)

[CR15] Baskerville R, Myers MD (2004). Special issue on action research in information systems: making IS research relevant to practice—foreward. In: MIS Quart.

[CR16] Baskerville R, Wood-Harper AT (1998). Diversity in information systems action research methods. In: European Journal of Information Systems.

[CR17] Beck K (2004). Extreme programming explained: embrace change.

[CR18] Boden M (1990). The creative mind - myths and mechanisms.

[CR19] Boehm B, Grunbacher P, Briggs RO (2001). Developing groupware for requirements negotiation: lessons learned. In: IEEE Software.

[CR20] Boehm B, Rombach HD, Zelkowitz MV (2005) Foundations of empirical software engineering: the legacy of victor R. Basili. Springer-Verlag Berlin Heidelberg

[CR21] Briand L (2012). Embracing the engineering side of software engineering. In: IEEE Software.

[CR22] Briand L, Bianculli D, Nejati S, Pastore F, Sabetzadeh M (2017). The case for context-driven software engineering research: generalizability is overrated. In: IEEE Software.

[CR23] Brooks F (1987). No silver bullet: essence and accidents of software engineering. In: Computer.

[CR24] Butt SA, Tariq MI, Jamal T, Ali A, Díaz Martinez JL, De-La-Hoz-Franco E (2019). Predictive variables for agile development merging cloud computing services. In: IEEE Access.

[CR25] Butt SA, Gochhait S, Andleeb S, Adeel M, Das S, Gochhait S (2021). Games features for health disciplines for patient learning as entertainment. Digital entertainment.

[CR26] Butt SA, Misra S, Anjum MW, Hassan SA, Przybyłek A, Miler J, Poth A, Riel A (2021). Agile project development issues during COVID-19. Lean and agile software development. LASD 2021. Lecture notes in business information processing.

[CR27] Campbell J, Kurkovsky S, Liew ChW, Tafliovich A (2016) Scrum and Agile Methods in Software Engineering Courses. In: 47th ACM Technical Symposium on Computing Science Education, Memphis, TN

[CR28] Cao L, Mohan K, Xu P, Ramesh B (2009). A framework for adapting agile development methodologies. In: Eur J Inf Syst.

[CR29] Carlson R (2013). Retrospectives are healthy.

[CR30] Caroli P, Caetano T (2016). Fun retrospectives - activities and ideas for making agile retrospectives more engaging.

[CR31] Cho JY, Lee E (2014). Reducing confusion about grounded theory and qualitative content analysis: similarities and differences. In: The Qualitative Report.

[CR32] Chua RY-J, Iyengar SS (2008). Creativity as a matter of choice: prior experience and task instruction as boundary conditions for the positive effect of choice on creativity. In: Journal of Creative Behavior.

[CR33] Clason DL, Dormody TJ (1994) Analyzing Data Measured By Individual Likert-Type Items. In: Journal of Agricultural Education, vol. 35(4). 10.5032/jae.1994.04031

[CR34] Coghlan D, Brannick T (2005). Doing action research in your own organization.

[CR35] Collier B, DeMarco T, Fearey P (1996). A defined process for project postmortem review. In: IEEE Software.

[CR36] Cook T, Campbell D (1979). Quasi-experimental design and analysis issues for field settings.

[CR37] Csikszentmihalyi M (1991). Flow: the psychology of optimal experience.

[CR38] Dal Sasso T, Mocci A, Lanza M, Mastrodicasa E (2017) How to gamify software engineering. In: 24th International Conference on Software Analysis, Evolution and Reengineering (SANER'17), Klagenfurt, Austria. 10.1109/SANER.2017.7884627

[CR39] Davis J, Zaner M, Farnham S, Marcjan C, McCarthy BP (2003) Wireless brainstorming: overcoming status effects in small group decisions. In: 36th Annual Hawaii International Conference on System Sciences, Big Island, HI. 10.1109/HICSS.2003.1173812

[CR40] Davison RM, Martinsons MG, Kock N (2004). Principles of canonical action research. In: Information Systems Journal.

[CR41] Daylamani-Zad D, Agius H, Angelides MC (2020). Reflective agents for personalisation in collaborative games. In: Artif Intell Rev.

[CR42] De Dreu CKW, Nijstad BA, Van Knippenberg D (2008). Motivated information processing in group judgment and decision making. In: Personality and Social Psychology Review.

[CR43] Defranco JF, Laplante PA (2017). A content analysis process for qualitative software engineering research. In: Innov Syst Softw Eng.

[CR44] DeLuca D, Gallivan JJ, Kock N (2008). Furthering information systems action research: a post-positivist synthesis of four dialectics. In: Journal of the Association for Information Systems vol.

[CR45] Denzin NK (1970). The research act in sociology: a theoretical introduction to sociological methods.

[CR46] Derby E, Larsen D (2006) Agile Retrospectives: Making Good Teams Great. Pragmatic Programmers

[CR47] Diehl M, Stroebe W (1987). Productivity loss in brainstorming groups: toward the solution of a riddle. In: Journal of Personality and Social Psychology.

[CR48] Dingsøyr T, Hanssen GK, Henninger S, Maurer F (2003). Extending agile methods: postmortem reviews as extended feedback. Advances in learning software organizations. LSO 2002. Lecture notes in computer science.

[CR49] Dingsøyr T, Mikalsen M, Solem A, Vestues K (2018) Learning in the large - an exploratory study of retrospectives in large-scale agile development. In: 19th International Conference, XP, Porto, Portugal. 10.1007/978-3-319-91602-6_13

[CR50] Drægert A, Petersen D (2016) ScrumBut in professional software development. MSc thesis, Department of Computer Science, Aalborg University

[CR51] Drury M, Conboy K, Power K (2012). Obstacles to decision making in agile software development teams. In: J Syst Softw.

[CR52] Dybå T, Maiden N, Glass R (2014). The reflective software engineer: reflective practice. In: IEEE Software.

[CR53] Dybå T, Dingsøyr T, Moe NB, Ruhe G, Wohlin C (2014). Agile Project Management. Software Project Management in a changing world.

[CR54] Dzieciątek A (2019). Analysis of the techniques for retrospectives in scrum projects.

[CR55] Easterbrook SM, Singer J, Storey MA, Damian D (2007) Selecting Empirical Methods for Software Engineering Research. In: F. Shull, J. Singer and D. Sjøberg (eds) Guide to Advanced Empirical Software Engineering, Springer

[CR56] Ellis S, Carette B, Anseel F, Lievens F (2014). Systematic reflection: implications for learning from failures and successes. In: Current Directions in Psychological Science.

[CR57] Elo S, Kyngäs H (2008). The qualitative content analysis process. In: Journal of Advanced Nursing.

[CR58] Eloranta V, Koskimies K, Mikkonen T (2016). Exploring ScrumBut — an empirical study of scrum anti-patterns. In: Information and Software Technology.

[CR59] Fredrickson BL (1998). What good are positive emotions?. In: Review of General Psychology.

[CR60] Fruhling AL, Tarrell AE (2008). Best practices for implementing agile methods: a guide for DOD software developers.

[CR61] Gaikwad PK, Jayakumar CT, Tilve E, Bohra N, Yu W, Spichkova M (2019). Voice-activated solutions for agile retrospective sessions. In: Procedia Computer Science.

[CR62] Garousi V, Borg M, Oivo M (2020). Practical relevance of software engineering research: synthesizing the community’s voice. In: Empir Software Eng.

[CR63] Gelperin D (2011) Increase requirements understanding by playing cooperative games. In: INCOSE International Symposium, Denver, CO

[CR64] Ghaisas S, Rose P, Daneva M, Sikkel K, Wieringa RJ (2013) Generalizing by similarity: Lessons learnt from industrial case studies. In: 1st International Workshop on Conducting Empirical Studies in Industry, San Francisco, CA, doi: 10.1109/CESI.2013.6618468

[CR65] Ghanbari H, Similä J, Markkula J (2015). Utilizing online serious games to facilitate distributed requirements elicitation. In: Journal of Systems and Softwar.

[CR66] Glass RL (1994). The software-research crisis. In: IEEE Software.

[CR67] Gonçalves L, Linders B (2014) Getting value out of agile retrospectives: a toolbox of retrospective exercises. Leanpub

[CR68] Gray D, Brown S, Macanufo J (2010) Gamestorming. A Playbook for innovators rulebreakers and changemakers. O'Reilly

[CR69] Griffin L (2021) Implementing Lean Principles in Scrum to Adapt to Remote Work in a Covid-19 Impacted Software Team. In: Przybyłek A., Miler J., Poth A., Riel A. (eds) Lean and Agile Software Development. LASD 2021. Lecture notes in business information processing, vol 408. Springer, Cham. 10.1007/978-3-030-67084-9_11

[CR70] Guckenbiehl P, Theobald S, Morisio M, Torchiano M, Jedlitschka A (2020). Impediment Management of Agile Software Development Teams. Product-focused software process improvement. PROFES 2020. Lecture notes in computer science.

[CR71] Gupta M, George JF, Xia W (2019). Relationships between IT department culture and agile software development practices: an empirical investigation. In: International Journal of Information Management.

[CR72] Hanslo R, Mnkandla E (2018) Scrum Adoption Challenges Detection Model: SACDM. In: 2018 Federated Conference on Computer Science and Information Systems (FedCSIS'18), Poznan, Poland, 2018. 10.15439/2018F270

[CR73] Highsmith J, Fowler M (2001). The agile manifesto. In: Softw Dev Mag.

[CR74] Hoda R, Babb J, Nørbjerg J (2013). Toward learning teams. IEEE Softw.

[CR75] Hoegl M, Parboteeah KP (2007) Creativity in innovative projects: How teamwork matters. In: J. Eng. Technol. Manag. 24, 1–2, 148–166, 2007. 10.1016/j.jengtecman.2007.01.008

[CR76] Hohmann L (2006). Innovation games: creating breakthrough products through collaborative play.

[CR77] Hsieh HF, Shannon SE (2005). Three approaches to qualitative content analysis. In: Qualitative Health Research.

[CR78] Hult M, Lennung S-Å (1980). Towards a definition of action research: a note and bibliography. In: Journal of Management Studies.

[CR79] International Institute of Business Analysis: A Guide to the Business Analysis Body of Knowledge v3 (BABOK Guide), Toronto, Canada (2015)

[CR80] Ilyés E (2019) Create your own agile methodology for your research and development team. In: 2019 Federated Conference on Computer Science and Information Systems (FedCSIS'19), Leipzig, Germany

[CR81] Jarzębowicz A, Sitko N, Wrycza S, Maślankowski J (2019). Communication and documentation practices in agile requirements engineering: a survey in polish software industry. Information systems: research, development, applications, education. SIGSAND/PLAIS 2019. Lecture notes in business information processing.

[CR82] Jeffries R (2013) Fractional Scrum, or “Scrum-But”. AgileAtlas,

[CR83] Jick TD (1979). Mixing qualitative and quantitative methods: triangulation in action. In: Administrative Science Quarterly.

[CR84] Jovanović M, Mesquida AL, Radaković N, Mas A (2016). Agile retrospective games for different team development phases. In: Journal of Universal Computer Science.

[CR85] Kaplan B, Duchon D (1988) Combining Qualitative and Quantitative Methods in Information Systems Research: A Case Study. In: MIS Quarterly, vol. (12)4, pp. 571–587

[CR86] Karau SJ, Williams KD (1993). Social loafing: a meta-analytic review and theoretical integration. J Pers Soc Psychol.

[CR87] Khanna D Experiential team learning in software startups. In: 19th international conference on agile software development: companion, 2018, Porto. Portugal. 10.1145/3234152.3314992

[CR88] Khorram F, Taromirad M, Ramsin R (2021) SeGa4Biz: Model-Driven Framework for Developing Serious Games for Business Processes. In: 9th International Conference on Model-Driven Engineering and Software Development, Online event. 10.5220/0010198801390146

[CR89] Kidder LH, Fine M, Mark MM, Shotland RL (1987). Qualitative and quantitative methods: when stories converge. New directions for program evaluation, N.35.

[CR90] Kontio J, Bragge J, Lehtola L, Shull F, Singer J, Sjøberg DIK (2008). The focus group method as an empirical tool in software engineering. Guide to advanced empirical software engineering.

[CR91] Korde R, Paulus PB (2017). Alternating individual and group idea generation: finding the elusive synergy. J Exp Soc Psychol.

[CR92] Krein JL, Prechelt L, Juristo N, Nanthaamornphong A, Carver JC, Vegas S, Knutson CD, Seppi KD, Eggett DL (2016). A Multi-Site Joint Replication of a Design Patterns Experiment Using Moderator Variables to Generalize across Contexts. In: IEEE Transactions on Software Engineering.

[CR93] Krivitsky A (2015). Agile retrospective Kickstarter.

[CR94] Kua P (2013). The retrospective handbook: a guide for agile teams.

[CR95] Küpper S, Pfahl D, Jürisoo K, Diebold P, Münch J, Kuhrmann M (2019) How has SPI changed in times of agile development? Results from a multi-method study. In: J Softw Evol Proc 31(11). 10.1002/smr.2182

[CR96] Lamoreux M (2005) Improving agile team learning by improving team reflections. In: Agile Development Conference, Denver, CO. 10.1109/ADC.2005.29

[CR97] Lamrani R, Abdelwahed EH, Chraibi S, Qassimi S, Hafidi M, El Amrani A, Rocha Á, Serrhini M, Felgueiras C (2017). Serious game to enhance and promote youth entrepreneurship. Europe and MENA cooperation advances in information and communication technologies. Advances in intelligent systems and computing.

[CR98] Lamrani R, Abdelwahed EH (2020). Game-based learning and gamification to improve skills in early years education. In: Computer Science and Information Systems Vol.

[CR99] Lehtinen TOA, Virtanen R, Viljanen JO, Mäntylä MV, Lassenius C (2014). A tool supporting root cause analysis for synchronous retrospectives in distributed software teams. In: Inf Softw Technol.

[CR100] Lehtinen TOA, Mäntylä MV, Itkonen J, Vanhanen J (2015). Diagrams or structural lists in software project retrospectives – an experimental comparison. In: Journal of Systems and Software.

[CR101] Lehtinen TOA, Itkonen J, Lassenius C (2017). Recurring opinions or productive improvements—what agile teams actually discuss in retrospectives. Empir Software Eng.

[CR102] Lenberg P, Wallgren Tengberg LG, Feldt R (2017). An initial analysis of software engineers’ attitudes towards organizational change. In: Empir Software Eng.

[CR103] Lin LH, Lin WH, Chen CY, Teng YF (2010) Playfulness and innovation - A multilevel study in individuals and organizations. In: 5th IEEE International Conference on Management of Innovation and Technology, Singapore

[CR104] Loeffler M (2017). Improving agile retrospectives: helping teams become more efficient.

[CR105] López-Martínez J, Juárez-Ramírez R, Huertas C, Jiménez S, Guerra-García C (2016) Problems in the adoption of agile-scrum methodologies: A systematic literature review. In: 4th International conference in software engineering research and innovation, Puebla, México. 10.1109/conisoft.2016.30

[CR106] Madeyski L, Kitchenham B (2017). Would wider adoption of reproducible research be beneficial for empirical software engineering research?. In: Journal of Intelligent & Fuzzy Systems.

[CR107] Madsen DB, Finger JR (1978). Comparison of a written feedback procedure, group brainstorming, and individual brainstorming. In: Journal of Applied Psychology.

[CR108] Goncalo JA, Mannix EA, Neale MA (2009). Creativity in groups.

[CR109] Marcinkowski B, Gawin B (2019). A study on the adaptive approach to technology-driven enhancement of multi-scenario business processes. In: Information Technology & People.

[CR110] Marek K, Wińska E, Dąbrowski W, Przybyłek A, Miler J, Poth A, Riel A (2021). The state of agile software development teams during the Covid-19 pandemic. Lean and agile software development. LASD 2021. Lecture notes in business information processing.

[CR111] Marijan D, Gotlieb A (2021) Industry-Academia Research Collaboration in Software Engineering: The Certus Model. In: Information and Software Technology 132. 10.1016/j.infsof.2020.106473

[CR112] Marshburn D (2018) Scrum Retrospectives: Measuring and Improving Effectiveness. In: SAIS 2018 Proceedings, https://aisel.aisnet.org/sais2018/26

[CR113] Mas A, Poth A, Sasabe S (2018) SPI with Retrospectives: A Case Study. In: 18th European Systems Software and Service Process Improvement and Innovation (EuroSPI), Bilbao, Spain

[CR114] Matthies Ch (2020) Playing with your project data in scrum retrospectives. In: 42nd International Conference on Software Engineering: Companion Proceedings (ICSE'20), Seoul, Korea. 10.1145/3377812.3382164

[CR115] Matthies Ch, Dobrigkeit F (2020) Towards Empirically Validated Remedies for Scrum Retrospective Headaches. In: 53rd Hawaii International Conference on System Sciences (HICSS’20), Honolulu, Hawaii

[CR116] Matthies C, Dobrigkeit F (2021) Experience vs Data: A Case for More Data-Informed Retrospective Activities. In: Przybyłek A., Miler J., Poth A., Riel A. (eds) Lean and Agile Software Development. LASD. Lecture notes in business information processing, vol. 408. Springer, Cham. 10.1007/978-3-030-67084-9_8

[CR117] Matthies C, Dobrigkeit F, Ernst A (2019) Counteracting Agile Retrospective Problems with Retrospective Activities. In: 19th European Systems Software and Service Process Improvement and Innovation (EuroSPI), Edinburgh, UK

[CR118] Matthies Ch, Dobrigkeit F, Hesse G (2020) Mining for Process Improvements: Analyzing Software Repositories in Agile Retrospectives. In: 42nd International Conference on Software Engineering Workshops (ICSEW'20), Seoul, Korea. 10.1145/3387940.3392168

[CR119] Mayring P (2014). Qualitative content analysis: Theoretical Foundation.

[CR120] McGonigal J (2011). Reality is broken.

[CR121] Mesquida AL, Jovanović J, Jovanović M, Mas A (2019). Agile software process improvement: a collaborative game toolbox. In: IET Software.

[CR122] Meyer J (2000) Evaluating action research. In: Age and Ageing, Vol. 29, Issue suppl_2: pp. 8–10, doi: 10.1093/oxfordjournals.ageing.a008104 10.1093/oxfordjournals.ageing.a008104 11109939

[CR123] Mich D, Ng YY (2020) Retrospective games in Intel Technology Poland. In: 15th Conference on Computer Science and Information Systems (FedCSIS'20), Sofia, Bulgaria, 2020. 10.15439/2020F62

[CR124] Michinov N (2012). Electronic brainstorming and Brainwriting. In: J Appl Soc Psychol.

[CR125] Miler J, Gaida P, Przybyłek A, Morales-Trujillo M (2020). Identification of the agile mindset and its comparison to the competencies of selected agile roles. Advances in agile and user-Centred software engineering. LASD 2019, MIDI 2019. Lecture notes in business information processing.

[CR126] Moe NB, Aurum A, Dybå T (2012). Challenges of shared decision-making: a multiple case study of agile software development. In: Inform Softw Technol.

[CR127] Mundra A, Misra S, Dhawale CA (2013) Practical Scrum-Scrum Team: Way to Produce Successful and Quality Software. In: 13th International Conference on Computational Science and Its Applications, Ho Chi Minh City, Vietnam, doi: 10.1109/iccsa.2013.25

[CR128] Nerur S, Mahapatra RK, Mangalaraj G (2005). Challenges of migrating to agile methodologies. In: Commun ACM.

[CR129] Neumann M, Bogdanov Y, Lier M, Baumann L, Przybyłek A, Miler J, Poth A, Riel A (2021). The Sars-Cov-2 pandemic and agile methodologies in software development: a multiple case study in Germany. Lean and agile software development. LASD 2021. Lecture notes in business information processing.

[CR130] Ng YY, Skrodzki J, Wawryk M, Przybyłek A, Morales-Trujillo ME (2020). Playing the sprint retrospective: a replication study. LASD/MIDI-2019. LNBIP.

[CR131] Nguyen L, Swatman PA (2003). Managing the requirements engineering process. In: Requirements Engineering.

[CR132] Nijstad BA, Paulus PB, Paulus PB, Nijstad BA (2003). (2003). Group creativity: common themes and future directions. Group creativity: innovation through collaboration (pp. 326–339).

[CR133] Nijstad BA, Stroebe W (2006). How the group affects the mind: a cognitive model of idea generation in groups. In: Personality and Social Psychology Review.

[CR134] Nijstad BA, Stroebe W, Diehl M, Paulus PB, Nijstad BA (2003). Cognitive stimulation and interference in idea-generating groups. Group creativity: innovation through collaboration (pp. 137–159).

[CR135] Nikitina N, Kajko-Mattsson M, Stråle M (2012) From Scrum to Scrumban: A Case Study of a Process Transition. In: International Conference on Software and System Process (ICSSP'12), Zurich, Switzerland

[CR136] von Neumann J, Morgenstern O (1944). Theory of games and economic behavior.

[CR137] Nunamaker JF, Applegate LM, Konsynski BR (1987). Facilitating group creativity: experience with a group decision support system. In: Journal of Management Information Systems.

[CR138] Olgun S, Yilmaz M, Clarke PM, O’Connor RV (2017) A Systematic Investigation into the Use of Game Elements in the Context of Software Business Landscapes: A Systematic Literature Review. In: 17th International Conference on Software Process Improvement and Capability Determination (SPICE), Spain, doi: 10.1007/978-3-319-67383-7_28

[CR139] de Oliveira Neto FG, Torkar R, Feldt R, Gren L, Furia CA, Huang Z (2019). Evolution of statistical analysis in empirical software engineering research: current state and steps forward. In: Journal of Systems and Software.

[CR140] Osborn AF (1957). Applied imagination.

[CR141] Özkan N, Gök MŞ, Köse BÖ (2020) Towards a Better Understanding of Agile Mindset by Using Principles of Agile Methods. In: 15th Conference on Computer Science and Information Systems (FedCSIS), Sofia, Bulgaria, doi: 10.15439/2020F46

[CR142] Pallot M, Le Marc C, Richir S, Schmidt C, Mathieu J (2012) Innovation gaming: an immersive experience environment enabling co-creation. In: M. Cruz-Cunha (Ed.), handbook of research on serious games as educational, business and research tools (pp. 1–24). Hershey, PA: Information Science Reference

[CR143] Paulus PB, Brown VR, Paulus PB, Nijstad BA (2003). Enhancing ideational creativity in groups: lessons from research on brainstorming. Group creativity: innovation through collaboration (pp. 110–136).

[CR144] Paulus PB, Brown VR (2007). Toward more creative and innovative group idea generation: a cognitive-social motivational perspective of brainstorming. In: Social and Personality Compass.

[CR145] Paulus PB, Dzindolet MT (1993). Social influence processes in group brainstorming. In: Journal of Personality and Social Psychology.

[CR146] Paulus PB, Dzindolet M (2008). Social influence, creativity and innovation. In: Social Influence.

[CR147] Paulus PB, Nijstad BA (2003) Group creativity: innovation through collaboration. Oxford Scholarship Online. 10.1093/acprof:oso/9780195147308.001.0001

[CR148] Paulus PB, Nijstad BA (2019). The Oxford handbook of group creativity and innovation.

[CR149] Petersen K, Wohlin C (2009) Context in industrial software engineering research. In: 3rd International Symposium on Empirical Software Engineering and Measurement (ESEM 2009), pp. 401–404

[CR150] Poth A, Riel A (2020) Quality Requirements Elicitation by Ideation of Product Quality Risks with Design Thinking. In: 28th International Requirements Engineering Conference (RE), Zurich, Switzerland. 10.1109/RE48521.2020.00034

[CR151] Potts C (1993). Software-engineering research revisited. In: IEEE Software.

[CR152] Prechelt L, Zieris F, Schmeisky H (2015) Difficulty Factors of Obtaining Access for Empirical Studies in Industry. In: IEEE/ACM 3rd International Workshop on Conducting Empirical Studies in Industry, Florence, Italy, doi: 10.1109/CESI.2015.11

[CR153] Przybylek A, Olszewski M (2016) Adopting collaborative games into Open Kanban. In: 2016 Federated Conference on Computer Science and Information Systems (FedCSIS'16), Gdansk, Poland. 10.15439/2016F509

[CR154] Przybyłek A, Kotecka D (2017) Making agile retrospectives more awesome. In: 2017 Federated Conference on Computer Science and Information Systems (FedCSIS'17), Prague, Czech Republic, 2017, 10.15439/2017F423

[CR155] Przybyłek A, Kowalski W (2018) Utilizing online collaborative games to facilitate Agile Software Development. In: 2018 Federated Conference on Computer Science and Information Systems (FedCSIS'18), Poznan, Poland, 2018, doi: 10.15439/2018F347

[CR156] Przybyłek A, Zakrzewski M (2018) Adopting Collaborative Games into Agile Requirements Engineering. In: 13th International Conference on Evaluation of Novel Approaches to Software Engineering (ENASE'18), Funchal, Madeira, Portugal, doi: 10.5220/0006681900540064

[CR157] Ramírez-Mora SL, Oktaba H, Patlán Pérez J (2020) Group maturity, team efficiency, and team effectiveness in software development: A case study in a CMMI-DEV Level 5 organization. In: J Softw Evol Proc 32(4). 10.1002/smr.2232

[CR158] Reason P, Bradbury H, Reason P, Bradbury H (2001). Introduction: inquiry and participation in search of world worthy of human aspiration. Handbook of action research: participative inquiry and practice (pp. 1–14).

[CR159] Reason P, Rowan J (1981). Human inquiry: a sourcebook of new paradigm research.

[CR160] Ringstad MA, Dingsøyr T, Brede Moe N (2011) Agile Process Improvement: Diagnosis and Planning to Improve Teamwork. In: 18th European Conference on Software Process Improvement (EuroSPI), Roskilde, Denmark

[CR161] Robbins NM, Heiberger RM (2011) Plotting Likert and other rating scales. In: JSM Proceedings of the Section on Survey Research Methods. American Statistical Association, pp. 1058–1066, Alexandria, VA

[CR162] Robey D, Ross JW, Boudreau MC (2002). Learning to implement enterprise systems: an exploratory study of the dialectics of change. J Manag Inf Syst.

[CR163] Robson C (2002). Real world research: a resource for social scientists and practitioners-researchers.

[CR164] Roden T, Williams B (2015). Fifty quick ideas to improve your retrospectives.

[CR165] Root-Bernstein RS (1989). Discovering: inventing and solving problems at the Frontiers of scientific knowledge.

[CR166] Rubin KS (2012). Essential scrum: a practical guide to the most popular agile process.

[CR167] Santanen EL, Briggs RO, de Vreede GJ (2000) The cognitive network model of creativity: a new causal model of creativity and a new brainstorming technique. In: 33rd Annual Hawaii International Conference on System Sciences, Maui, HI, doi: 10.1109/HICSS.2000.926895

[CR168] Santos PSMD, Travassos GH (2009) Action research use in software engineering: An initial survey. In: 3rd International Symposium on Empirical Software Engineering and Measurement, Lake Buena Vista, FL, 2009. doi: 10.1109/ESEM.2009.5316013

[CR169] Sawyer K (2007) Group genius: the creative Power of collaboration. Cambridge, MA

[CR170] Seaman CB (1999). Qualitative methods in empirical studies of software engineering. In: IEEE Transactions on Software Engineering.

[CR171] Sjøberg DIK, Dyba T, Jørgensen M (2007a) The future of empirical methods in software engineering research. In: Future of Software Engineering, Minneapolis, MN. 10.1109/fose.2007.30

[CR172] Spiegler SV, Heinecke C, Wagner S (2021). An empirical study on changing leadership in agile teams. In: Empir Software Eng.

[CR173] Stettina CJ, Offerman T, De Mooij B, Sidhu I (2018) Gaming for Agility: Using Serious Games to Enable Agile Project & Portfolio Management Capabilities in Practice. In: 2018 IEEE International Conference on Engineering, Technology and Innovation (ICE/ITMC), Stuttgart, Germany, doi: 10.1109/ICE.2018.8436384

[CR174] Schön EM, Winter D, Escalona MJ, Thomaschewski J, Baumeister H, Lichter H, Riebisch M (2017). Key challenges in agile requirements engineering. Agile processes in software engineering and extreme programming. XP 2017. Lecture notes in business information processing.

[CR175] Schwaber K (2004). Agile Project Management with scrum.

[CR176] Schwaber K, Sutherland J (2017) The Scrum Guide — The Definitive Guide to Scrum: The Rules of the Game. www.scrumguides.org

[CR177] Schwaber K, Sutherland J (2020) The Scrum Guide — The Definitive Guide to Scrum: The Rules of the Game. www.scrumguides.org

[CR178] Shin Y (2014). Positive group affect and team creativity: mediation of team reflexivity and promotion focus. Small Group Res.

[CR179] Sjøberg DIK, Dyba T, Jørgensen M (2007b) The future of empirical methods in software engineering research. In: Future of Software Engineering, Minneapolis, MN, doi:10.1109/fose.2007.30

[CR180] Spradley JP (1980). Participant observation.

[CR181] Staron M (2020) Action research in software engineering. Springer, Cham. 10.1007/978-3-030-32610-4

[CR182] Sternberg RJ (1999). Handbook of creativity.

[CR183] Susman G (1983) Action research: a sociotechnical systems perspective. In: Beyond Method: Strategies for Social Research (Morgan G, Ed), pp 95–113, Sage, Newbury Park

[CR184] Susman GI, Evered RD (1978). An assessment of the scientific merits of action research. Administrative Science Quarterly vol.

[CR185] Sutherland J (2015). Scrum: the art of doing twice the work in half the time.

[CR186] Taylor DW, Berry PC, Block CH (1958). Does group participation when using brainstorming facilitate or inhibit creative thinking?. Administrative Sciences Quarterly.

[CR187] Theobald S, Prenner N, Krieg A, Schneider K, Morisio M, Torchiano M, Jedlitschka A (2020). Agile leadership and agile management on organizational level - a systematic literature review. Product-focused software process improvement. PROFES 2020. Lecture notes in computer science.

[CR188] Trujillo MM, Oktaba H, González JC (2014) Improving Software Projects Inception Phase Using Games: ActiveAction Workshop. In: 9th International Conference on Evaluation of Novel Approaches to Software Engineering (ENASE'14), Lisbon, Portugal

[CR189] Trujillo MM, García-Mireles GA, Maslova P (2018) What Can Go Wrong in a Software Project? Have Fun Solving It. In: 2018 Federated Conference on Computer Science and Information Systems (FedCSIS'18), Poznan, Poland

[CR190] Üsfekes Ç, Tüzün E, Yılmaz M, Macit Y, Clarke P (2019) Auction-based serious game for bug tracking. In: IET Softw, Vol. 13(5), pp. 386-392, 2019. 10.1049/iet-sen.2018.5144

[CR191] VersionOne: 14th Annual State of Agile Report. Tech. report (2020)

[CR192] Wawryk M, Ng YY (2019) Playing the Sprint Retrospective. In: 14th Federated Conference on Computer Science and Information Systems, Leipzig, Germany, 2019, doi: 10.15439/2019F284

[CR193] Westera W (2017). How people learn while playing serious games: a computational modelling approach. In: Journal of Computational Science.

[CR194] Wieringa R, Daneva M (2015). Six strategies for generalizing software engineering theories. In: Science of Computer Programming.

[CR195] Wohlin C (2013) Software engineering research under the lamppost. In: 8th International Joint Conference on Software Technologies, Reykjavík, Iceland

[CR196] Yilmaz M (2013) A software process engineering approach to understanding software productivity and team personality characteristics: an empirical investigation. Phd thesis, Dublin City University, 2013

[CR197] Yilmaz M, O’Connor RV (2016). A Scrumban integrated gamification approach to guide software process improvement: a Turkish case study. In: Technical Gazette.

[CR198] Yilmaz M, O'Connor R, Mora M (2016). Improving social aspects of the software development process: games. Gamification and Related Approaches In: Journal of Universal Computer Science.

[CR199] Yilmaz M, O’Connor RV, Colomo-Palacios R, Clarke PM (2019). Guest editorial: gamification and persuasive games for software engineering. In: IET Software.

[CR200] Yin RK (2018). Case study research and applications: design and methods.

[CR201] Yu X, Petter S (2014). Understanding agile software development practices using shared mental models theory. In: Information and Software Technology.

[CR202] Zagal JP, Rick J, Hsi I (2006) Collaborative games: Lessons learned from board games. In: Simulation & Gaming, vol. 37(1), pp. 24–40, March 2006

